# Advanced Technologies for Large Scale Supply of Marine Drugs

**DOI:** 10.3390/md23020069

**Published:** 2025-02-07

**Authors:** Henar Martínez, Mercedes Santos, Lucía Pedraza, Ana M. Testera

**Affiliations:** 1 Department of Organic Chemistry, School of Engineering (EII), University of Valladolid (UVa), Dr. Mergelina, 47002 Valladolid, Spain; henar.martinez@uva.es (H.M.); msantos@uva.es (M.S.); 2 G.I.R. Computational Chemistry Group, Department of Physical Chemistry and Inorganic Chemistry, Science Faculty, University of Valladolid (UVa), Paseo de Belén 7, 47011 Valladolid, Spain; 3 G.I.R. Bioforge, University of Valladolid (UVa), CIBER-BBN, Paseo de Belén 19, 47011 Valladolid, Spain; 4 Department of Organic Chemistry, Science Faculty, University of Valladolid (UVa), Paseo de Belén 7, 47011 Valladolid, Spain; lucia.pedraza@estudiantes.uva.es

**Keywords:** total synthesis, aquaculture, chemoenzymatic synthesis, fermentation, ex vivo biosynthesis, cell factory, production strategies

## Abstract

Marine organisms represent a source of unique chemical entities with valuable biomedical potentialities, broad diversity, and complexity. It is essential to ensure a reliable and sustainable supply of marine natural products (MNPs) for their translation into commercial drugs and other valuable products. From a structural point of view and with few exceptions, MNPs of pharmaceutical importance derive from the so-called secondary metabolism of marine organisms. When production strategies rely on marine macroorganisms, harvesting or culturing coupled with extraction procedures frequently remain the only alternative to producing these compounds on an industrial scale. Their supply can often be implemented with laboratory scale cultures for bacterial, fungal, or microalgal sources. However, a diverse approach, combining traditional methods with modern synthetic biology and biosynthesis strategies, must be considered for invertebrate MNPs, as they are usually naturally accumulated in only very small quantities. This review offers a comprehensive examination of various production strategies for MNPs, addressing the challenges related to supply, synthesis, and scalability. It also underscores recent biotechnological advancements that are likely to transform the current industrial-scale manufacturing methods for pharmaceuticals derived from marine sources.

## 1. Introduction

Marine ecosystems have a high diversity of living organisms compared to terrestrial ecosystems providing a vast and largely untapped reservoir of potential pharmaceutical resources [1,2,3] and numerous resources for human nutrition [4]. The biological diversity in the ocean is estimated to range from 0.7 to 1.0 million eukaryotic species, but with millions more prokaryotic and viral taxa, with up to 95% of marine organisms being estimated to be microbial [1]. Along with such biological diversity, a vast chemical diversity has also been recorded [1,5].

The high marine biodiversity is the consequence of the wide range of unique conditions in which organisms survive (temperature, pressure, nutrient variations, light intensity, and free oxygen) which has induced species to develop mechanisms of evolution and adaptation not necessary for terrestrial organisms. Therefore, marine natural products (MNPs) usually have unique structural scaffolds, biological modes of action [6], and have an important role as hit or lead compounds in drug discovery [2,6,7].

Marine natural products exhibit distinctive structural characteristics that set them apart from their synthetic counterparts. These compounds typically feature a higher proportion of sp^3^-hybridized carbon atoms and an increased number of stereogenic centers that contribute to their complex three-dimensional architectures. The molecular frameworks of marine-derived substances often incorporate a greater abundance of fused aliphatic ring systems, while simultaneously displaying a reduced frequency of aromatic moieties compared to synthetic drugs. In terms of elemental composition, MNPs generally contain high levels of carbon and hydrogen atoms, along with oxygen and nitrogen. This characteristic can be attributed to the saturated nature of many marine compounds [8]. A particularly noteworthy feature of these compounds is the notable presence of halogen atoms, predominantly chlorine and bromine, which can be attributed to two primary factors: the high concentration of halides in the marine environment and the presence of specialized halogenating enzymes that have evolved in marine organisms [7].

The distinctive molecular architecture of marine natural products contributes significantly to their diverse biological activities and potential pharmaceutical applications [9,10]. These structural features, which differ markedly from those of traditional synthetic drug candidates, underscore the importance of marine natural products in the ongoing search for novel therapeutic agents and bioactive compounds.

More than 42,000 compounds (peptides, fatty acids, polysaccharides, alkaloids, terpenoids, polyketides, non-ribosomal peptides and glycosides, etc.) have been isolated from marine micro- and macroorganisms including, but not limited to, fungi, bacteria, microalgae, macroalgae, sponges, corals, mollusks, and tunicates, with hundreds of new MNPs being discovered every year [11,12]. Historically, the largest number of marine-derived secondary metabolites, including some of the most promising drug candidates, have been produced by marine invertebrates [2]. However, in recent years, the total number of new marine natural products (MNPs) reported annually indicates that marine fungi have emerged as the most prolific source of new MNPs. There has been a decreasing trend in the reporting of metabolites from cyanobacteria, mangrove-associated fungi, and algae, while reports of sponge- and cnidarian-derived MNPs have shown an increase [11].

Marine-based pharmaceuticals have started to impact modern pharmacology [1,13,14,15] and numerous literature data from recent years indicate heightened attention to the high potential of marine organisms for the biomedical and pharmaceutical industries [3,16]. About a quarter of the isolated MNPs, so far, have exhibited significant potential as therapeutic agents for addressing conditions like cancer, drug-resistant bacteria, viral diseases, and immune suppressive disorders [2] reflecting interesting biological activities including antiproliferative, antimicrobial, anti-inflammatory, anticoagulation, analgesic, neuroprotective, and cardioprotective properties [1,3,7,17,18,19].

However, despite that rich biodiversity, biologically active substances from marine organisms are not always available in the required amount [3] and few products derived from the sea have made it through to industrial production compared to those arising from terrestrial environments. Currently, over 30 drugs derived from marine natural products (MNPs) have been produced on a large scale through classical chemical synthesis, enabling clinical trials that resulted in their approval by various health organizations worldwide. Additionally, six of these drugs are in advanced stages of development [3,20]. They are currently used to cure/treat some of the main medical pathologies including cancer, tumor growth, leukemia, cardiovascular diseases, bronchial asthma, Alzheimer’s disease, hypertonic disease, disorders in the processes of blood circulation, autoimmune diseases, pain syndromes, and viral infections [1,17,21,22].

Approximately one hundred MNPs enter preclinical development annually. The discovery of new compounds with promising biomedical potential and innovative mechanisms of action shows no signs of slowing, raising hopes that many more drugs will be derived from MNPs in the near future [23,24,25]. Efficient progress through clinical trials stage is a lengthy process, with limitations due to lack of sustainable supply, structural complexity and poor pharmacokinetic properties that must be overcome by full synthetic manufacture, new procurement technologies including the optimization of fermentation processes, the encapsulation of drugs in nanoparticles, and, as a very successful approach, their use as payloads in antibody drug conjugates (ADCs) [3,7].

Consequently, whether for preclinical and clinical development or marketing, it seems important to be able to anticipate efficient processes of sustainable supply for promising MNP-derived drugs, requiring quantities in a gram range [20], and even more in the perspective of a future commercialization where annual demands usually reach several kilograms [3,26,27,28].

Production strategies for marine macroorganism natural products normally rely on harvesting or culturing coupled with extraction procedures as the only alternative for their production [29]. With respect to marine invertebrates such as sponges and tunicates, although concentrations of the promising MNPs in themselves may be sufficient for their chemical characterization and preliminary activity evaluation, aquaculture of invertebrates is far from providing a sustainable supply for pre-clinical and clinical trials [20,30].

Alternative production methods, such as total chemical synthesis, semi-synthesis, invertebrate and symbiont cell culture, fermentation, and several hybrid strategies have to be developed to meet the growing demand for marine-derived pharmaceuticals [14,20,27,31,32,33].

Especially in the case of new MNPs of great potential but displaying structures far too complex for a profitable production by chemical synthesis (not possible or economically prohibitive for drug development), alternative biotechnological approaches could be considered [3]. Recent advances have facilitated the semi-synthetic production of complex pharmaceutical compounds and commodity chemicals. Biocatalytic methods have become recognized tools to enhance efficiency and selectivity in fine chemical processes, enabling access to bioactive compounds. In particular, in vitro multi-enzyme synthesis demonstrates the potential of biocatalytic approaches to produce complex natural compounds through a minimal number of enzymatic steps, addressing the significant synthetic challenges often associated with marine natural products [4]. On the other hand, developments in synthetic biology and biotechnology such as metabolomics and metagenomics, molecular networking, genetic engineering, genome sequencing and mining, advanced screening systems, and combinatorial biosynthesis offer promising solutions to overcome production obstacles [34,35]. These cutting-edge technologies enable the engineering of enhanced microbial platforms for the heterologous biosynthesis of MNPs [31,36,37], though scaling these methods from the laboratory to industrial production remains a significant challenge.

In this review, we aim to explore the current state of large-scale production methods for marine-derived pharmaceuticals. We will discuss the strategies employed to overcome the challenges of supply, synthesis and scalability, as well as highlight recent advances in biotechnological innovations that are poised to revolutionize the contemporary landscape of industrial-scale manufacturing techniques for pharmaceuticals of marine origin. In some cases, non-marine-derived compounds are included as illustrative examples to showcase the versatility and applicability of different techniques, particularly when these strategies are relevant to the synthesis of marine natural products. By addressing these issues, we seek to provide a comprehensive overview of the potential and limitations of marine natural products in the pharmaceutical industry. Articles selected for this review prioritize recent peer-reviewed studies examining gram-scale production of these bioactive compounds which remains challenging due to their often-complex molecular structures and limited availability from natural sources. This ensures the inclusion of the most relevant and up-to-date findings that we hope will meet the needs of researchers and industry stakeholders.

## 2. Aquaculture

The production of complex MNPs biosynthesized by marine invertebrates differ from those currently used for several drugs or their precursors extracted from terrestrial medicinal plants. Random sampling of sessile marine organisms directly from their natural environment is not acceptable. Beyond the ecological impact, the total mass naturally available is often insufficient to meet industrial demand. More importantly, such practices pose significant risks to biodiversity [38].

### 2.1. Aquaculture Methods: Mariculture vs. Captive Breeding

To address these challenges, aquaculture of drug-producing marine invertebrates has been explored since the 1980s, drawing analogies from advances in monoculture-based production of plant-derived pharmaceuticals such as the anticancer vinca alkaloids (Vincristine, Vinblastine, and Vinorelbine) from the *Madagascar periwinkle* (*Catharanthus roseus*) or the antimalarial artemisinin from *Artemisia annua* [39]. Two primary aquaculture methods have been developed: mariculture (or in situ aquaculture) and captive breeding (or ex situ aquaculture). Both approaches have their own advantages and disadvantages with regard to MNP production [40,41].

In situ techniques or mariculture take place in the natural habitat of the marine organisms. While facing specific challenges due to climatic variations and environmental threats, it also represents a simple, low-cost methodology that relies entirely on the natural environment to provide light and nutrients essential for the growth of marine species. In contrast, ex situ aquaculture (captive breeding) is carried out in controlled systems where growth conditions can be optimized [42]. The production of MNPs, initially identified in cnidarians, sponges, mollusks, bryozoans, ascidians, and echinoderms, depends not only on the biosynthetic pathways of marine metazoan species but also on associated microorganisms [1]. However, it also faces challenges associated with the limited availability and structural complexity of MNPs which makes large-scale production have low yields and slow growth rates when cultured outside their natural environment [20] and can alter the composition of symbiotic microorganisms, essential to producing marine metabolites [42]. Furthermore, predation poses a significant obstacle in mariculture systems, as exemplified by the study of Page et al., where nudibranch infestations severely impacted sponge biomass and metabolite yields, highlighting the ecological complexities of scaling up production [43].

### 2.2. Aquaculture-Derived Compounds: Clinical Development

Despite the challenges, some efforts have been made to produce specific compounds through aquaculture. Several of these marine-derived compounds have advanced to clinical trials, highlighting the potential of MNPs despite ongoing challenges. Some efforts have been made to produce specific compounds through aquaculture [44,45]. Didemnin, isolated from the tunicate *Trididemnum solidum*, was among the first marine compound to enter phase II trials for cancer treatment, although its toxicity precluded therapeutic use. The compound was initially extracted from 53.4 g of freeze-dried tunicate material resulting in 5.5 g of crude extract, from which Didemnin was isolated with a low yield of approximately 0.45% (24.9 mg per 5.5 g of crude extract) [46]. Aplidin (Plitidepsin (**21**)), a less toxic analog derived from *Aplidium albicans*, but predominantly synthesized by total synthesis [47], has since replaced it and received orphan drug status for treatment of multiple myeloma and acute lymphoblastic leukemia in 2004 [48,49]. Bryostatin, derived from *Bugula neritina*, required 13 metric tons of biomass to produce just 18 g of compound, underscoring the scalability challenges [50]. Although supercritical CO_2_ extraction methods have been attempted to improve Bryostatin extraction, significant scalability issues remain [50]. Similarly, Dolastatin 10, an anti-tumoral peptide from *Dolabella auricularia*, served as a model for synthetic analogs despite its limited clinical efficacy [51]. These production methods, involving both “in situ” and “ex situ” aquaculture, have ultimately been abandoned due to their low profitability and sustainability.

### 2.3. Innovative Technologies for Sustainable Marine Bioprospecting

To address the sustainability challenges in marine bioprospecting, several innovative technologies have been developed, each offering unique approaches to capture bioactive molecules directly in the marine habitat while minimizing environmental impact.

One such advancement is “Somartex”, a system that employs solid-phase extraction to selectively isolate and concentrate compounds, enabling their collection in sufficient quantities for chemical characterization and even potential gram-scale production. By eliminating the need to harvest entire organisms, Somartex represents a groundbreaking approach to resolving the sustainability issues associated with traditional extraction methods. The patented system works by deploying solid-phase extraction technology in open marine environments, allowing compounds to be captured directly from the water surrounding marine invertebrates in their natural habitat. This process minimizes ecological disturbance and preserves marine biodiversity, addressing a critical concern in the field of marine bioprospecting. Using the Somartex technology, 160 mg of crude methanol extract was obtained during aquarium experiments, enabling the isolation of bioactive compounds such as crambescidin 359 (1.5 mg), crambescidin acid (1.1 mg), and crambescidin 401 (1.1 mg), among others, in milligram-scale quantities. Furthermore, the system has demonstrated the feasibility of integrating sustainable practices into the discovery and production of marine-derived pharmaceuticals. In the initial trials, Somartex successfully captured guanidine alkaloids from marine sponges without causing harm to the organisms. This achievement highlights the technology’s potential to meet growing demand for marine natural products (MNPs) while reducing the environmental impact of collection practices. Additionally, the versatility of the Somartex system suggests its applicability to a wide range of marine organisms and compounds, paving the way for broader use in pharmaceutical research and commercial production. As marine biodiversity continues to be a critical resource for drug discovery, such innovations are vital for ensuring long-term sustainability in the field [42].

Similarly, the I-SMEL (In situ Marine moleculE Logger) system has recently demonstrated significant advances in the in situ collection of marine natural products (MNPs) in sponge-dominated Mediterranean environments. This device captures and concentrates marine metabolites directly from seawater, eliminating the need to harvest or disturb the marine organisms that produce these valuable compounds. Unlike previous methods, the I-SMEL is manageable by a single SCUBA diver and allows for the non-destructive capture of exometabolites released by organisms, preserving both the sponges and their ecological surroundings [52]. By operating directly in the organisms’ natural habitat, I-SMEL bypasses some of the key limitations of traditional methods, such as low yields and environmental degradation caused by biomass collection. The system uses solid-phase extraction (SPE) cartridges to filter controlled volumes of seawater, retaining compounds such as brominated alkaloids, such as Aerothionin, Purealidin L, Aerophobin-1, and terpenoids, from sponges like *Aplysina cavernicola* and *Spongia officinalis*. These compounds are known for their bioactive properties, including antimicrobial, anti-inflammatory, and antitumoral activities. Remarkably, this approach allows the continuous trapping of exometabolites, which are naturally released by marine organisms into the surrounding seawater, without causing harm to the sponges or disrupting their ecological roles. In recent experimental trials, the I-SMEL was subjected to three specific studies: the first explored the average chemical composition of the marine environment by filtering 10 L of seawater; the second profiled exometabolites from different sponge species; and the third examined temporal variations in metabolic secretions from the same organism [52]. While the method has not yet reached gram-scale production, it offers a promising, non-destructive, and sustainable alternative to conventional extraction methods. By reducing reliance on biomass harvesting and fostering a deeper understanding of how marine species interact with their environment through metabolite release, the I-SMEL system represents a pivotal step forward in the field of marine biotechnology. Future refinements and scaling efforts could make this technology a viable tool for obtaining marine-derived compounds for pharmaceutical and industrial applications for obtaining marine-derived compounds for pharmaceutical and industrial applications [53].

Recently, the Small Molecule In Situ Resin Capture (SMIRC) approach has emerged as a culture-independent method for microbial natural product discovery. Unlike Somartex and I-SMEL, which primarily focus on metabolites from larger marine organisms like sponges, SMIRC targets microbial diversity, capturing compounds directly from the environment without requiring cultivation. This method uses adsorbent resins to isolate natural products from bacteria, phytoplankton, and other microorganisms, bypassing the need for cultivation or the activation of silent biosynthetic gene clusters. SMIRC represents a significant shift from traditional microbial natural product discovery, which has long been constrained by challenges such as the re-isolation of known compounds, the difficulty of culturing many microbial species, and the limited activation of biosynthetic pathways under laboratory conditions. By capturing metabolites directly from their natural environment, SMIRC expands access to previously untapped chemical diversity, uncovering new compounds and carbon skeletons. Moreover, SMIRC can be modified to enhance in situ microbial growth, leveraging natural environmental cues to activate biosynthetic pathways and increase the yield of novel compounds [54].

Although Somartex, I-SMEL, and SMIRC all employ in situ techniques, each system is tailored to address specific challenges in marine bioprospecting, highlighting their complementary roles in sustainability and compound discovery. Somartex is designed for selective extraction and scalability, making it particularly effective for industrial applications that require large quantities of specific compounds. It utilizes solid-phase extraction (SPE) technology to isolate and concentrate bioactive molecules directly from the water surrounding marine invertebrates, enabling gram-scale production while minimizing ecological disturbance. For example, the system has successfully extracted guanidine alkaloids from marine sponges without harming the organisms, demonstrating its potential for sustainable pharmaceutical production. In contrast, I-SMEL focuses on the non-invasive capture of naturally released exometabolites, offering a sustainable method for studying marine organisms in their natural habitats. The system’s portability allows for precise collection of compounds such as brominated alkaloids and terpenoids from seawater, preserving marine biodiversity while enabling ecological and pharmaceutical research. Unlike Somartex, which targets industrial scalability, I-SMEL excels in non-destructive sampling, facilitating deeper understanding of marine species’ interactions with their environments. Meanwhile, SMIRC addresses an entirely different niche by targeting microbial diversity and capturing natural products directly from bacteria, phytoplankton, and other microorganisms without requiring cultivation. By using adsorbent resins, SMIRC bypasses traditional challenges in microbial natural product discovery, such as the re-isolation of known compounds and the difficulty of activating biosynthetic pathways in laboratory settings. This method expands access to untapped chemical diversity, uncovering new compounds and carbon skeletons while offering the flexibility to enhance microbial growth in situ. Together, these systems exemplify the innovation and versatility required to sustainably harness marine biodiversity for pharmaceutical and industrial applications, with each method providing distinct advantages tailored to specific research and production needs [42,53,54].

The emergence of these technologies, “Somartex”, I-SMEL, and SMIRC, offer promising solutions to these obstacles, providing sustainable methods to produce marine compounds without negatively impacting biodiversity. These advancements highlight the potential to meet pharmaceutical demand while addressing environmental concerns. Nevertheless, further research and development are critical to optimize these technologies, scale up production, and ensure the consistent supply of MNPs required by the pharmaceutical industry.

## 3. Chemical Total Synthesis and Semi-Synthesis

The aquaculture methods described so far, in situ and ex situ, may prove to be promising in the future, especially if they go hand in hand with biotechnological advances. However, they have not yet found application in large-scale MNPs production and have been temporarily shelved due to their low profitability and sustainability [42]. The natural alternative could be the total chemical synthesis of MNPs that would allow industrial-scale production. One of the reasons for their non-scalability lies in their complex structure, which makes global chemical synthesis routes unfeasible in most cases [20]. The unique and extreme conditions endured by marine organisms and to which they have adapted has led to the production of marine compounds with particularly complex chemical structures with the presence, among others, of halogenated atoms as well as a high proportion of chiral centers, sp^3^-hybridized carbons, and condensed aliphatic rings, as mentioned in the introduction [8]. Of the many isolated MNPs, more than a quarter have been shown to have great potential as therapeutic agents. However, the bottleneck in their application lies in obtaining them in the quantities required for clinical and preclinical trials, and even for their subsequent commercialization.

There have been many products initially obtained from marine organisms that were unsuccessfully attempted to be supplied at scale through total chemical syntheses. However, this methodology brings with it an additional advantage: obtaining valuable intermediate compounds and the development of derivatives with more manageable, less complex, properties, which would later be used in further studies. As established with eribulin mesylate (Halaven^®^), advances in chemical synthesis continuously stretch the boundaries of our capacities for manufacturing pharmaceuticals with increasingly complex structures [55]. Among the many attempts at total synthesis of eribulin, the one developed by Nicolau [56] demonstrates the extent to which the limits of total synthesis of natural products have been exceeded in order to reach industrial scale.

This section will focus on the description of the synthesis of natural products of pharmacological interest, initially discovered and isolated from marine micro and macroorganisms, whose medical application has been demonstrated and, finally, have been approved by the FDA. We will focus on those commercial compounds that, in most examples, have been prepared on an industrial scale from strategies based on total organic synthesis. Semi-synthetic routes of special relevance for their importance in the supply of MNPs on an industrial scale will also be considered. We will review those syntheses of special synthetic value that, although they did not allow industrial scale-up, have allowed us to advance in the search for those total chemical syntheses that did allow production at scale [57].

Pharmaceutical MNPs encompass chemical compounds of different classes and with very different structural complexities. For the latter reason, the process and cost of production will also be very different from one compound to another. In this section we will describe the total synthesis of nucleosides of interest such as Cytarabine (**1**), Vidarabine (**8**), and Nelarabine (**4**) which, in general, are simpler than those we will describe leading to depsipeptides such as Plitidepsin (**21**). We will finish by describing the total syntheses of much more complex compounds of interest such as macrolides like Bryostatin-1 or alkaloids like Trabectedin (**29**). We will focus on the most interesting routes from the synthetic point of view but, fundamentally, we will describe their total synthesis applicable to industrial scale-up.

### 3.1. Synthesis of Nucleosides: Cytarabine, Nelarabine, and Vidarabine

#### 3.1.1. Cytarabine

The nucleoside Cytarabine (**1**), or Cytosine arabinoside (ara-C), was discovered and isolated for the first time in the 1940s by Werner Bergmann from the marine sponge *Cryptotethya crypta*. This so-called spongonucleoside has a 2′-OH in the arabino configuration. Several nucleosides, including spongonucleosides such as Cytarabine, were prepared in the late 1950s in the belief that they could interfere with DNA replication and cause cancer cell death, but were later shown to be able to inhibit DNA synthesis in vitro and, by the 1960s, were shown to be cytotoxic to cancer cells [58]. In fact, Cytarabine (**1**) is converted to its triphosphate form inside the cell and competes with cytidine for incorporation into DNA. The sugar moiety of Cytarabine hinders the turnover of the DNA molecule, halting DNA replication during the S phase of the cell cycle, making it a compelling drug for rapidly dividing cells, such as those seen in cancer. Cytarabine (**1**) rapidly evolved through testing in rodent animal models and clinical trials to gain FDA approval as a cancer therapy and is now specifically used for the treatment of acute myeloid leukemia [59] and lymphomas.

**Scheme 1:**
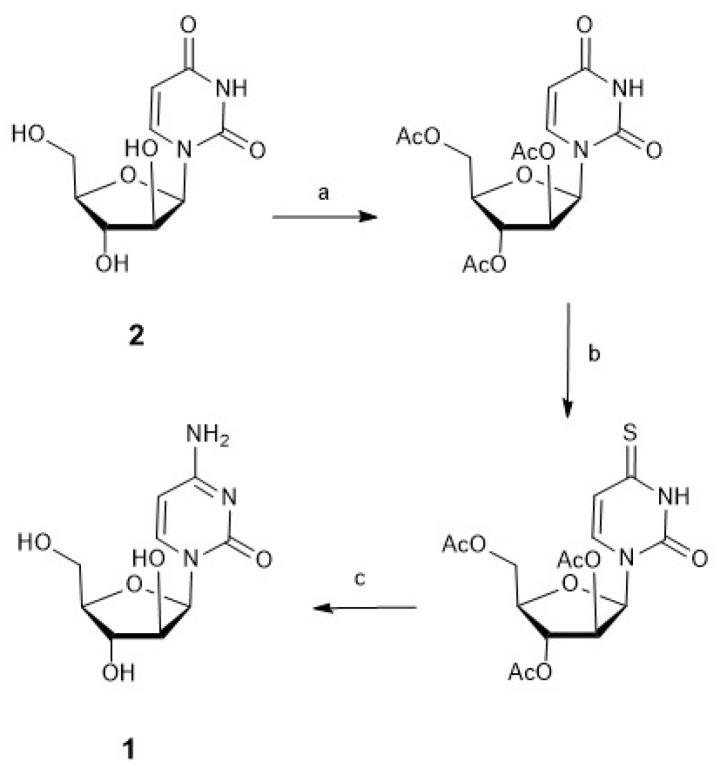
Synthesis of Cytarabine (**1**). Reagents and conditions: (**a**) (CH_3_CO)_2_O; (**b**) P_2_S_5_; (**c**) NH_3_/CH_3_OH [60].

The recognized chemical synthesis of Cytarabine (**1**) is carried out from uracil arabinoside (**2**) in a three-step process (Scheme 1), which initially involves acetylation of the hydroxyl groups; in a second step, the formation of a thiocarbonyl group from the carbonyl group at position 4 of the pyrimidine ring by reaction with phosphorus pentasulphide; and, finally, a third step involving the substitution of the mercapto group with an amino group by ammonia in methanol, with simultaneous hydrolysis of the acetyl groups, leading directly to Cytarabine (**1**) [60].

**Scheme 2:**
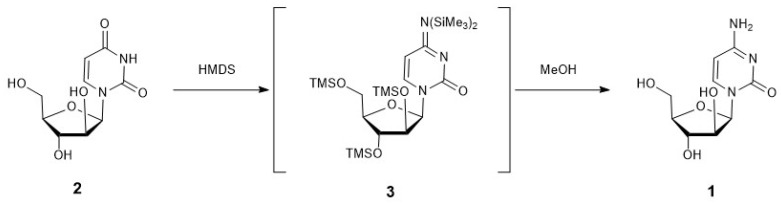
Industrial-scale synthesis of Cytarabine (**1**) patented by Chunhua et al. in 2010 [60].

The industrial-scale chemical synthesis of Cytarabine (**1**) was patented in 2010 using a synthetic chemical process (Scheme 2) that has the advantages of starting from a cheap and available raw material, giving a high yield, low cost, with simple reaction conditions and environmental protection [60]. The synthetic route starts, as in the synthesis described above, from uracil arabinoside (ara-U, **2**), which is reacted with hexamethyldisilazane (HDMS) in excess (1:2 to 1:10 ratio) at elevated pressure (5–20 atm) and temperature of 70–160 °C for 10–80 h. After vacuum concentration, once excess HDMS is removed, the intermediate **3** is solved in a suitable solvent such as methanol which results in deprotection of the amine and hydroxyl groups to afford Cytarabine (**1**) in a process with only two steps which produces Cytarabine (**1**) in high yield (75–90% yield and total production of 51 g to 65 g from 80 g of ara-U, **2**). This yield was improved in the pilot plant scale-up, which allowed its production on an industrial scale [60].

#### 3.1.2. Nelarabine

Nelarabine (2-amino-9-β-D-arabinosyl-6-methoxy-9H-guanine) (**4**) is the water-soluble prodrug of 9-β-D-arabinofuranosylguanine, ara-G, via conversion in the serum by endogenous adenosine deaminase [61]. The medical use of Nelarabine (**4**) is due to its 10-fold greater solubility than ara-G [62,63]. Although ara-G was discovered in the 1960s, its clinical development occurred after the 1970s, based on studies of the rare autosomal disorder causing purine nucleoside phosphorylase deficiency resulting in T-cell lymphopenia without concomitant decrease in B-cell populations [64].

Ara-G is converted by cellular kinases into the active metabolite ara-G-5′-triphosphate which, as a substrate for DNA polymerases, is incorporated into DNA causing inhibition of DNA replication and leading to cell death by apoptosis [65]. Studies in a murine model of T-cell acute lymphoblastic leukemia (ALL) in which the bone marrow, contaminated with malignant T-cells and treated ex vivo with the drug, showed that Nelarabine (**4**) treatment purged the bone marrow of malignant T-cells without irreversible toxicity to haematopoietic stem cells [66]. As a result, Nelarabine (**4**) was approved by the FDA in 2005 for the treatment of relapsed T-cell acute lymphocytic leukemia and relapsed T-cell lymphoblastic lymphoma [67]. In April 2021, the FDA approved a Nelarabine (**4**)-based injectable therapy, SH-11, developed by Shorla Pharma Limited, for the treatment of patients with T-cell leukemia [68].

**Scheme 3:**
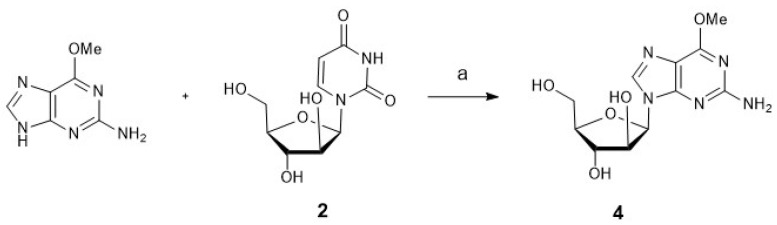
Synthesis of Nelarabine (**4**). Reagents and conditions: (**a**) 10 mM K_3_PO_4_, n-PrOH, H_2_O, pH 6.75, uridine phosphorylase, purine nucleoside phosphorylase, 37 °C, 26 days, 17% yield [69].

Nelarabine (**4**) synthesis was first described by Krenitsky et al. through a trans-glycosylation process catalyzed by two enzymes, uridine phosphorylase and purine nucleoside phosphorylase, providing only the N9, β-anomer Nelarabine (**4**), in a low yield of 17% but using a process that takes only 26 days (Scheme 3) [69].

**Scheme 4:**
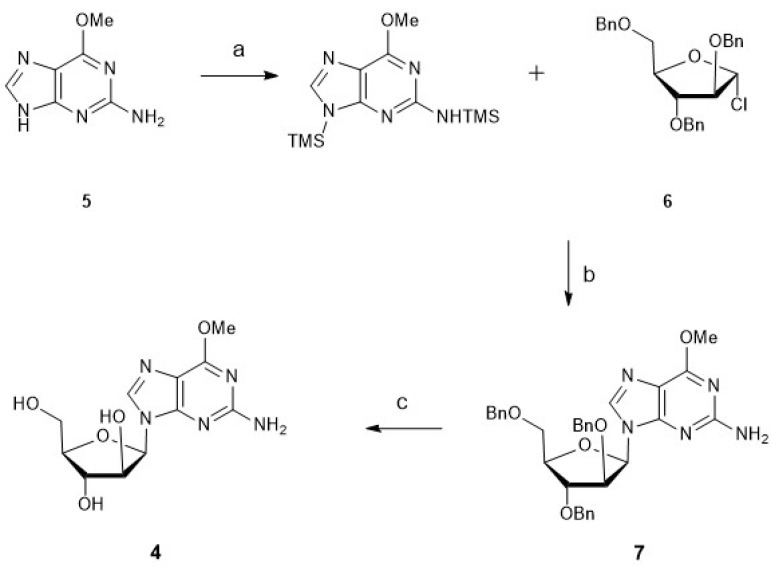
Industrial-scale synthesis of Nelarabine (**4**). Reagents: (**a**) HDMS/(NH_4_)_2_SO_4_; (**b**) (1) TMSOTf/CH_2_Cl_2_ and (2) NaHCO_3_/H_2_O; (**c**) BCl_3_/CH_2_Cl_2_ [70].

The gram-scale synthesis of Nelarabine (**4**) was first patented by Zong et al. The synthetic method comprises a first step of protecting the amino groups of 2-amino-6-methoxypurine, (**5**), with hexamethyldisilazane. The suitably protected product reacts with 2,3,5-tri-*O*-benzyl-1-chlorine-arabinose, (**6**), in a subsequent step catalyzed by trimethylsilyl fluorosulfonate to give 2-amino-6-methoxy-9-β-D-(2′,3′,5′-tri-*O*-benzyl-arabinosyl)purine, (**7**). The subsequent deprotection of the benzylated hydroxyl groups with boron trichloride and purifications both chromatographically and by recrystallization resulted in the production of Nelarabine (**4**) in amounts on the order of 10 g from 16 g of the starting purine (Scheme 4) [70].

#### 3.1.3. Vidarabine

**Figure 1:**
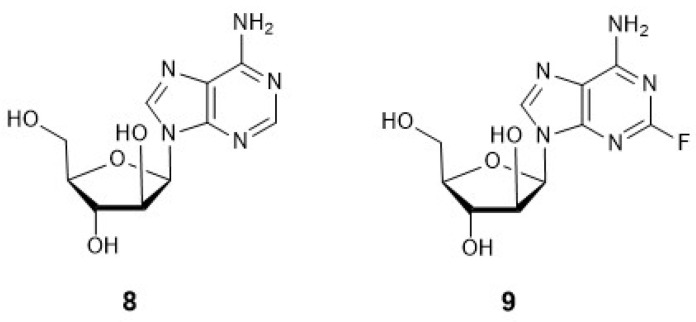
Structures of Vidarabine (**8**) and Fludarabine (**9**).

Vidarabine, 9-β-D-Arabinofuranosyladenine (ara-A) (**8**) together with Cytarabine (ara-C) (**1**) and protamine sulfate, was one of the first compounds of marine origin to be used in clinical use and was formally approved by the US FDA in 1976 as an ophthalmic ointment [71]. Although it was initially considered antiviral and even tested for its anticancer effect [72], its clinical use was ruled out as the therapeutically active concentration of the drug was not reached due to its relatively low solubility and high body clearance. In fact, the drug is rapidly deaminated to its inactive arabinosyl hypoxanthine form by adenosine deaminase, restricting its clinical use to limited pathological conditions [73,74]. In order to avoid adenosine deaminase mediated deactivation, Vidarabine (**8**) was initially replaced by Fludarabine (**9**) which carries, in addition, a fluorine at 2-position of the adenine (Figure 1) [75].

#### 3.1.4. Fludarabine Phosphate

The fluorinated derivative of Vidarabine (**8**), Fludarabine (**9**), was found to be poorly soluble and more complex to formulate, so it was replaced by the derivative Fludarabine-5-monophosphate (Fludara or fludarabine phosphate (**10**)). Fludarabine phosphate (**10**) once explored in clinical trials, was approved by the FDA in 1991 and is a commonly used drug for chronic lymphoid leukemia and hairy cell leukemia [76]. Fludarabine (**9**) is metabolized to its dephosphorylated form (F-ara-A) before being transported into the cell, and inside the cell it is rephosphorylated to F-ara-adenosine-triphosphate (Fara-ATP) by adenylate kinase and subsequently by nucleoside diphosphate kinases [77,78]. Once accumulated in an adequate cellular concentration, Fara-ATP is cytotoxic as it competes with other nucleotides in the synthesis of nucleic acids [77].

**Scheme 5:**
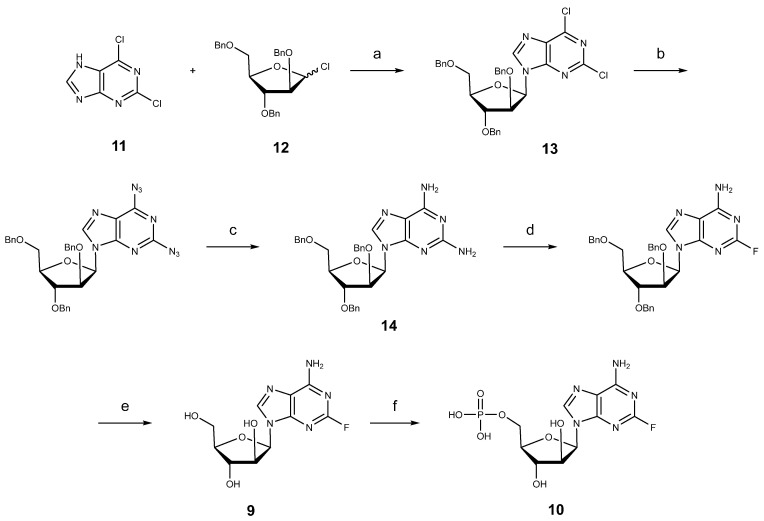
Synthesis of Fludarabine (**9**) and Fludarabine-5-monophosphate (**10**). Reagents and conditions: (**a**) Hg(CN)_2_, CaSO_4_, MeNO_2_, reflux, 3 h, 11%; (**b**) NaN_3_, EtOH, H_2_O, 1 h, 98%; (**c**) Pd/C, H_2_, EtOH, rt, 6 h, 75%; (**d**) HBF_4_, NaNO_2_, CHCl_3_, −10 °C, 40 min, 36%; (**e**) Na, NH_3_, 34%; (**f**) POCl_3_, PO(OEt)_3_, 0 °C, 3.5 h, 96% [81].

Fludarabine (**9**) was first synthesized in 1969 by Montgomery and Hewson via the glycosylation reaction between 2,6-dichloropurine (**11**) and chlorosugar (**12**), although the desired β-anomer (**13**) was obtained in a low yield of 11%. The next stage of nucleophilic substitution on the halogenated groups by reaction with sodium azide and subsequent hydrogenation catalyzed by Pd/C gave rise to diamino purine (**14**). The Balz–Schiemann reaction selectively obtained the purine derivative fluorinated at carbon 2 which, after deprotection of the hydroxyl groups, gives rise to Fludarabine (**9**) (Scheme 5) [79]. The yield of this Fludarabine (**9**) synthesis was improved years later by Montgomery when he used the acetylated derivative of 2,6-diaminopurine [80], leading to a gram-scale synthesis of Fludarabine phosphate (**10**) by phosphorylation reaction with trimethylphosphate and phosphoryl chloride (Scheme 5) [81]. Among the numerous routes designed for its synthesis, most recently, Kshirsagar et al. achieved kilogram-scale synthesis (yield of 2.5 kg) by innovating the hydroxyl debenzylation step by transfer hydrogenation, using ammonia formate as an in situ hydrogen donor, producing Fludarabine (**9**) at 99.8% purity in 90–95% yield [82].

**Scheme 6:**
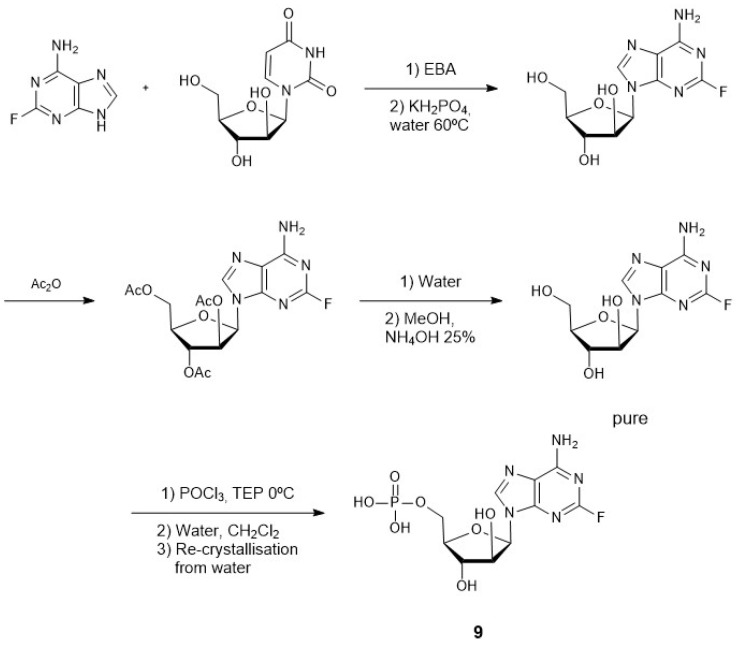
Industrial-scale synthesis of Fludarabine (**9**) patented by Farina et al. in 2003 [83].

We would like to highlight the industrial-scale synthesis of Fludarabine (**9**) obtained by initially reacting 2-fluoroadenine with 9-β-D-arabinofuranosyl-uracil (Ara-U) in the presence of *Enterobacter aerogenes* (EBA). Simply, the subsequent acetylation step of hydroxyl groups allows its purification by crystallization whose subsequent hydrolysis with methanol and ammonia allows obtaining Fludarabine (**9**) which, once purified by recrystallisation, has HPLC purity higher than 99% (Scheme 6). Phosphorylation of Fludarabine (**9**) according to a conventional methodology with trimethylphosphate and phosphoryl chloride occurs in high yield thanks to temperature control both in the reaction (−10 °C) and in the subsequent recrystallisation from water at 0 °C. In the patented example, a production of 64 g of Fludarabine (**9**) was obtained from 150 g of 2-fluoroadenine in a process that does not require chromatographic purification [83].

**Scheme 7:**
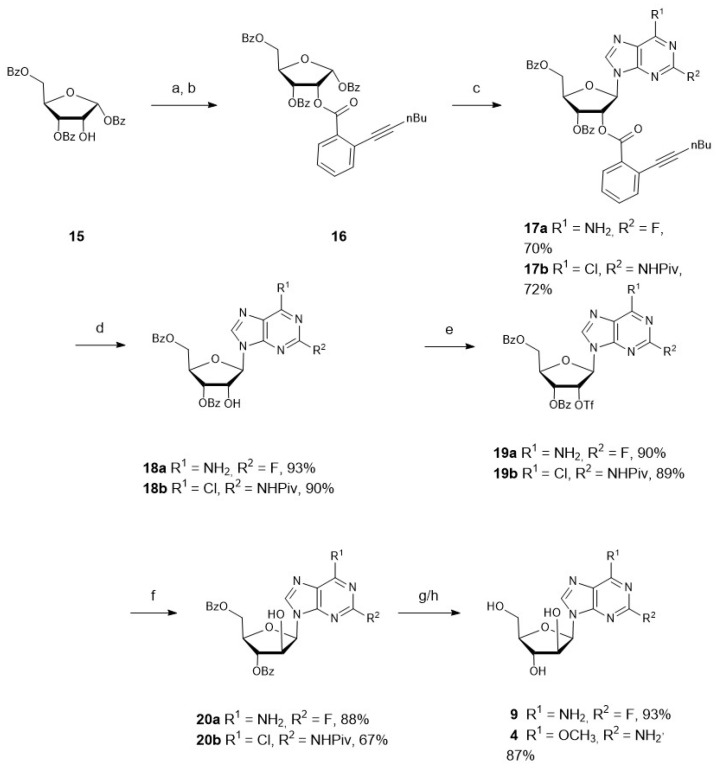
Synthesis of Fludarabine (**9**) developed by Xiao et al. Reagents and conditions: (**a**) 2-iodobenzoyl chloride, Py, ice-bath; (**b**) 1-hexyne, Pd(Ph_3_P)_2_Cl_2_, CuI, Et_3_N, THF, 50 °C, 5 h; (**c**) nucleobase, BSA, TMSOTf, CH_3_CN, 80 °C, 4 h; (**d**) Ph_3_PAuOTFA (5 mol%), EtOH, H_2_O, CH_2_Cl_2_, r.t., 5 h; (**e**) Tf_2_O, Py, CH_2_Cl_2_, −20 °C, 5 h; (**f**) KNO_2_, 18-crown-6, DMF, r.t., 24 h; (**g**) NH_3_, MeOH, 40 °C, 6 h; (**h**) NaOH, MeOH, 40 °C, 3 h. Reproduced with permission from Xiao, Q., Synthesis (Stuttg); 2020 [84].

Xiao et al. have developed a practical synthetic method to prepare Fludarabine (**9**) with a total yield of 35% overall, a potential application for future preparation on an industrial scale. The synthesis starts from 1,3,5-tri-*O*-benzoyl-α-D-ribose (**15**), a commercial reagent available in large quantities at a very reasonable price (1 kg, 150 US dollars), which is transformed into its *ortho*-alkyne benzoyl ester (**16**), in a two-step process (esterification with 2-iodobenzoyl chloride and subsequent Sonogashira reaction with 1-hexyne) with a good overall yield of 74% in a process applicable at 100 g scale without purification by column chromatography (Scheme 7) [84]. The subsequent Vorbrüggen glycosylation reaction with the silyl nucleobase, afforded the nucleoside (**17a**) with 70% yield and complete β-selectivity. Once it has performed its mission of assisting glycosylation, the *ortho*-alkyne benzoyl ester group is removed and, after reversion of the configuration of 2′-OH and corresponding deprotection, Fludarabine (**9**) was obtained in high total yield (35%) and purity (Scheme 7). This process has also been applied to the synthesis of Nelarabine (**4**), starting from the same commercial sugar and carried out using the Vorbrüggen glycosylation with the 2-amino-6-methoxy derivative of the silyl nucleobase, obtaining Nelarabine, (**4**), with a total yield of 24% and complete β-selectivity. This new synthetic procedure provides an ingenious alternative to the synthesis of other 2′-modified nucleosides on a large scale [84].

### 3.2. Synthesis of Depsipeptide: Plitidepsin

Plitidepsin (**21**) or Dehydrodidemnin B, marketed by PharmaMar (Madrid, Spain) under the trade name Aplidin^®^, is a cyclic depsipeptide, i.e., a cyclic peptide with one or more ester bonds instead of peptide bonds, which was first isolated from a Mediterranean marine tunicate (*Aplidium albicans*). Aplidin exhibits antiviral, immunosuppressive, and antitumor properties [85] and has been shown to induce apoptosis of leukemia cell by interruption of the cell cycle at the G1 and G2/M phases [86].

Aplidin^®^ has been manufactured by total synthesis in several steps involving the binding of six-amino acid to form the main backbone and with side chains composed of three amino acids: *N*-Me-*L*-Tyrosine and (*R*)-*N*-Me-*D*-Leucine linked to pyruvyl-*L*-Proline. Plitidepsin (**21**) has also been produced by total synthesis from suitably protected constituent amino acids, which in an iterative multi-step assembly process using a total solid-phase approach results in the composition of the skeleton that will subsequently undergo a solid-phase macrocyclization step and the deprotection and cleavage of global groups from the resin to produce the cyclodepsipeptide, although the process has numerous steps, low overall yield (3.3–4.8%), and is time-consuming to obtain (2 months) [87].

**Figure 2:**
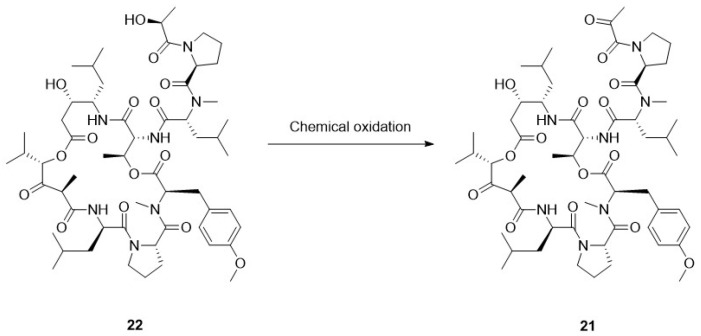
Single-step synthesis of Plitidepsin (**21**) in 90% yield from Dimemnin B (**22**) produced by *Tristella mobilis* L17. Reproduced with permission from Tang, X., bioRxiv; 2023 [88].

Plitidepsin (**21**) is a member of the didemnin family and, specifically, has a very similar structure to Didemnin B (**22**), differing only in that the lactate residue in Didemnin B (**22**) is present in the oxidized pyruvate form for Plitidepsin (**21**). Recently, Tang et al. have developed a new single-step synthetic route to convert Didemnin B (**22**) to Plitidepsin, (**21**) (Figure 2). The process consisted of the regioselective oxidation of Didemnin B (**22**) to Plitidepsin (**21**) using the radical catalyst 2,2,6,6-tetramethylpiperidinyloxy (TEMPO), with sodium hypochlorite (NaClO) as the stoichiometric oxidant, yielding pure Plitidepsin (**21**) in an overall yield of 90%. This synthetic route to Plitidepsin (**21**) includes the microbial synthesis of Didemnin B (**20**), whose production has been optimized using *Tristella mobilis* L17, together with the chemical synthesis mentioned above in a novel, economical and efficient approach that may prove promising for future industrial production [88].

**Scheme 8:**
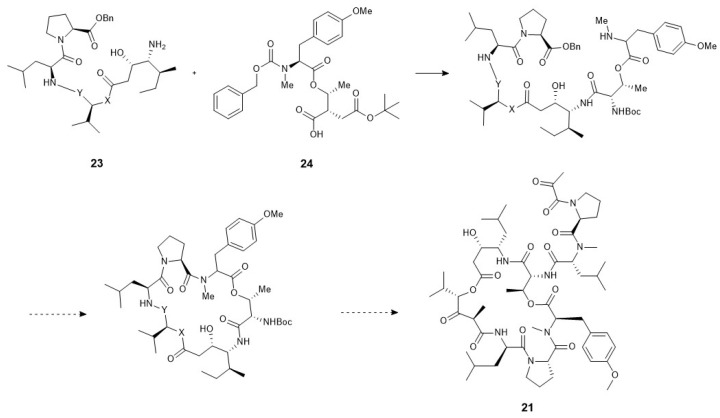
Synthesis of Plitidepsin (**21**) patented by Pharma Mar S.A. in 2001 [89].

The preparation of Aplidin^®^ on an industrial scale was patented in 2001 by PharmaMar (Madrid, Spain) as a PCT [89]. The synthetic process initially involves the preparation of the Didemnin fragment leading to the main backbone of the chain, by convergent coupling reaction between two fragments of Didemnin (**23**) and (**24**) (Scheme 8). These fragments had previously been obtained by successive joining of the amino or hydroxy acid subunits, essentially by successive amidation/esterification reactions, considering the corresponding necessary functional group protection/deprotection steps. The joining of the two fragments and subsequent cyclisation gives rise to the macrocycle present in the Aplidin^®^.

The preparation of the side chain is carried out by subsequent attachment of the amino acid leucine by amidation, followed by attachment to this side chain of pyruvyl-*L*-Proline. The whole process involves the conscientious protection and deprotection of the functional groups involved in each of the reactions. The technology protected in PharmaMar invention patent includes the preparation of both Aplidin^®^ and its derivatives and can also be used in the synthesis of a wide number of didemnins [89].

### 3.3. Synthesis of Macrolide: Bryostatin-1

The bryostatins are a group of marine macrolides found in marine invertebrate animals. Among the family of bryostatins, Bryostatin-1 (**25**) is the flagship due to its considerable biological activities. Numerous preclinical and clinical studies have demonstrated the promising potential of Bryostatin-1 (**25**) in treating cancer [90], Alzheimer’s disease [91], multiple sclerosis [92], or neurological disorders [93]. The biological activity of Bryostatin-1 (**25**) lies in its ability to bind to protein Kinase C (PKC), a protein involved in a variety of processes such as proliferation, differentiation, migration, and cell survival. After binding, Bryostatin-1 (**25**) induces the self-phosphorylation and activation of PKCs rapidly by promoting its membrane translocation. Given the important biological activity observed, it was necessary to develop an efficient procedure to obtain it. As mentioned in the aquaculture section, an efficient process, finally economically profitable with respect to those described above, was designed for the large-scale isolation of this macrocyclic lactone from the marine bryozoan *Bugula neritina* L. (*Bugulidae*). Starting from large quantities of this invertebrate (37.8 ton wet), using an isolation process guided by “A phorbol dibutyrate (PDBu) receptor binding assay” and Good Manufacturing Practices (GMPs), they were able to obtain a total mass of 18 g in four batches and in a process that they completed in 10 working months [50]. After this first supply of the compound, this material was used for formulation studies, preclinical toxicology, and clinical trials in cancer patients [50,94].

**Scheme 9:**
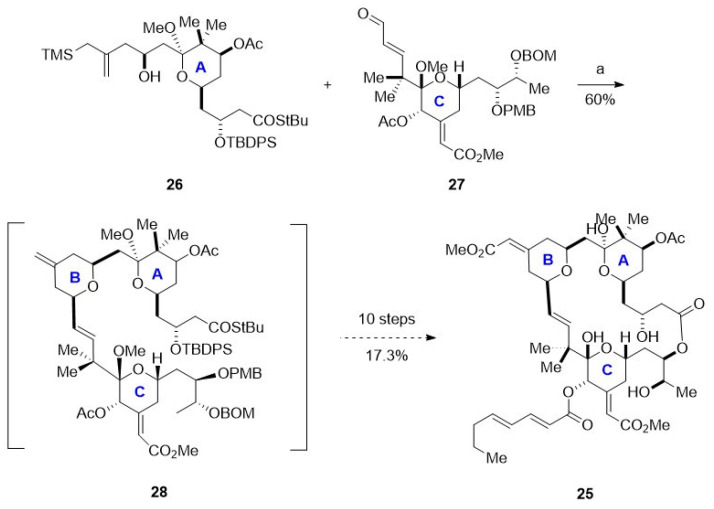
Synthesis of Bryostatin-1 (**25**) from the macrolide intermediate (**28**), in a 10-step process with a yield of 17.3%. A-ring allylsilane (**26**) and enal C-ring (**27**) union by Prins reaction gives rise to the formation of the B ring of the macrolide intermediate. Reagents: (**a**) TMSOTf, Et_2_O, −78 °C, 5 h (60% yield) [95].

One of the most promising syntheses of Bryostatin-1 (**25**) was carried out by Keck [95] in late 2010, using highly innovative synthetic reaction technologies developed both in his laboratory and that of his colleague Rainier. In this synthesis, Keck initially succeeded in preparing the A-ring allylsilane (**26**) (process of 19 steps) and the C-ring (**27**) in a 16-step process that includes Rainier’s titanium-mediated RCM reaction as the decisive step to obtain the C-ring of the macrolide. A-ring (**26**) subsequently binds to the enal C-ring (**27**) of the southern hemisphere of the macrolide, in a novel Lewis acid-mediated intermolecular Prins union performed at a low temperature to give B-ring in intermediate (**28**) of the macrolide. The intermediate macrolide (**28**) is subsequently subjected to a process consisting of 10 synthetic steps which are developed with a yield of 17.3% from the macrolide, and which finally yields Bryostatin-1 (**25**) (Scheme 9). It is a total synthesis with good stereoselectivity, which exploits new synthetic methodologies of the Keck group and with reduction by more than 20 step less than the original synthesis of Bryostatin-1 (**25**) developed by Hale et al. [96].

It is finally Wender’s group that in 2017 carried out a total synthesis of Bryostatin-1 (**25**) on a multigram scale, allowing a production of 20 g per year [97], providing the solution to the problem of both the clinical and research supply of Bryostatin-1 (**25**), as well as that of its analogs. The process is a step economical convergent synthesis, which proceeds in only 29 steps, with the longest synthetic sequence consisting of 19 steps. The total synthesis runs at 4.8% overall yield (>80% average yield per step) and results in the production of gram quantities of the compound (>2 g), with the possibility of scale-up if further production is required for clinical trials.

**Scheme 10:**
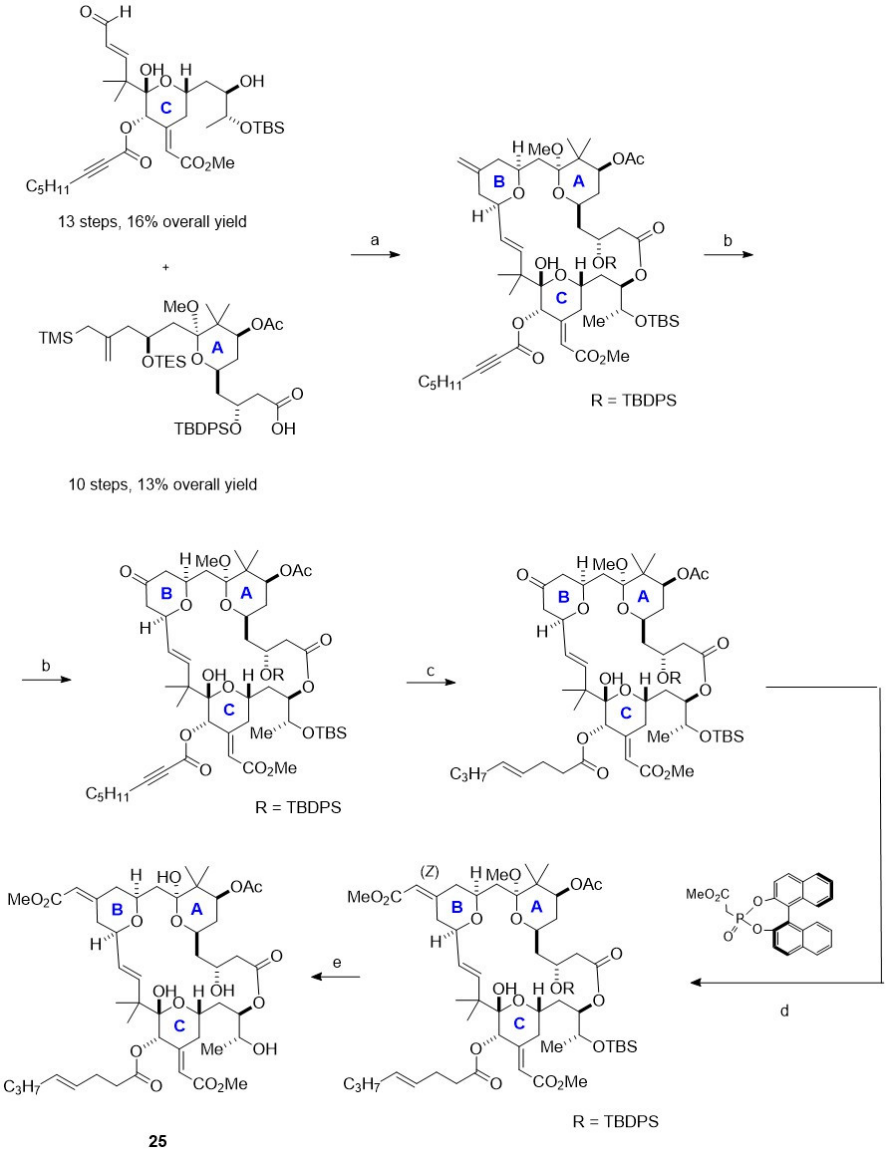
Synthesis of Bryostatin-1 (**25**) at multigram scale. The enal C-ring and the allylsilane A-ring are joined via Yamaguchi esterification followed by Prins macrocyclization with simultaneous formation of the B-ring. Reagents and conditions: (**a**) 1.-Yamaguchi esterification (82%), 2.-PPTS, MeOH (76%); (**b**) O_3_ (80%)/PPh_3_ (90%); (**c**) PPh_3_, MePhOH (90%); (**d**) NaHDMS (92%) (11:1, Z:E); (**e**) HF-pyr (80%*)* [98].

The synthesis of Bryostatin-1 (**25**) is designed in such a way that the two rings, A and C, are initially produced in parallel synthesis and then joined by a Yamaguchi esterification followed by Prins macrocyclization with simultaneous formation of the B ring. After four subsequent steps, Bryostatin-1 (**25**) is obtained, although one of these last steps must be emphasized: the complex isomerization of alkynoate to dienoate carried out according to the method described by Rychnovsky (90% yield) [98]. This convergent synthetic route has the additional advantage of synthetic versatility as both the subunits and the various intermediates can be modified to address the synthesis of new bryostatin analogs and derivatives (Scheme 10).

### 3.4. Synthesis of Alkaloid: Trabectedine

The ecteinascidins are marine natural products consisting of two or three linked tetrahydroisoquinoline subunits and an active carbinolamine group and are known to have potent antiproliferative activity against various tumor cells. The lead compound is Trabectedin (ET-743) (**29**), marketed as Yondelis^®^, which was the first marine antitumor drug approved in the European Union for treating soft tissue sarcoma (STS). Trabectedin (**29**) shows potent antiproliferative activity due to its tendency to bind to the minor groove of DNA, preferentially with the GC-rich triplets and subsequently forming covalent adducts with the N-2 position through the carbinolamine group, which interferes with DNA transcription processes [99]. Together with other Ectenaiscidins and its analog Lurbinectedin (ET-736, Zepzelca™), sea squirt *Ecteinascidia turbinata* was initially discovered in the Caribbean. The low amounts present in natural ascidian, with contents of only 0.5 to 4.0 µg per g of freshly harvested biomass, together with the fact that it can be isolated below 1.0 µg g^−1^, and in a complex chemical extraction process, with high economic costs and environmental impact, made it necessary to develop a synthetic process to produce the compound.

**Scheme 11:**
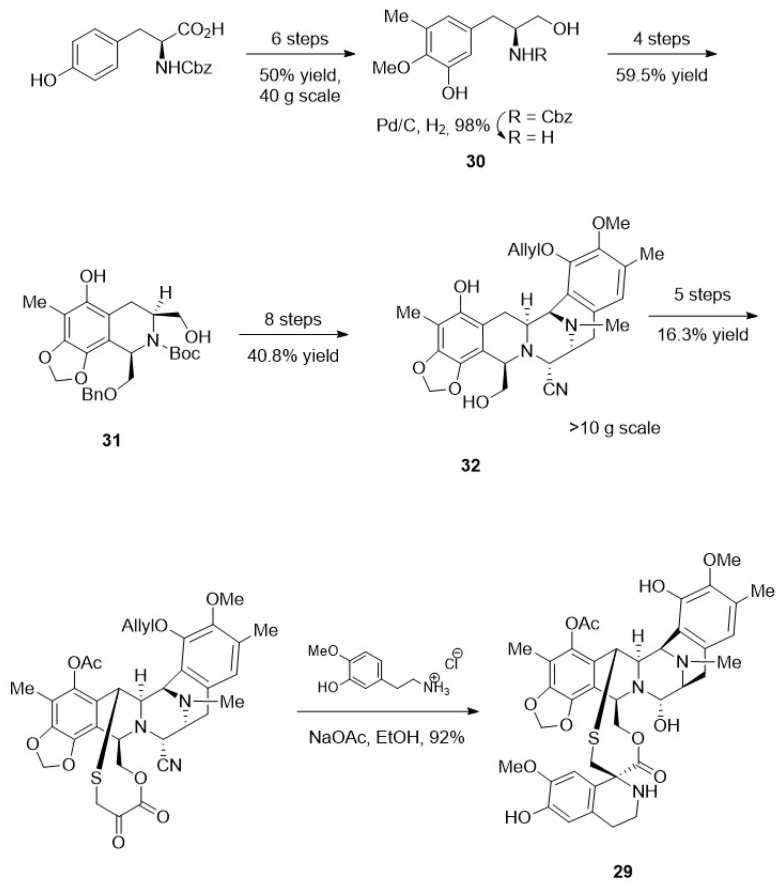
Synthesis of Trabectedin (**29**) at gram scale [107].

The first total synthesis of ET-743 (**29**) was developed by Corey in 1996 in an enantio and stereocontrolled, short, convergent process that achieved the 32-step synthesis of Ecteinascidin 743 (**29**), from sesamol in high yield [100]. This brilliant total synthesis has served as the basis for new synthetic routes and is described in great detail, explaining the configurational and mechanistic aspects of each stage of the process, in the article by Carmen Cuevas [101]. But neither this total synthesis nor the synthetic improvements developed by Martinez and Corey in 2000 for the production of the aminonitrile intermediate, in which an increase in production yield from 55 to 87% is achieved [102], have allowed ET-743 (**29**) to be obtained on an industrial scale. The groups of Fukuyama [103], Zhu [104], Danishefsky [105], and Williams [106] have developed elegant total syntheses of ET-743 (**29**) that remain far from scalable preparation due, among other reasons, to the use of expensive reagents that are difficult to access and the high number of synthetic steps (about 40–60 steps). He et al. have described an efficient and scalable total synthesis of ET-743 (**29**) from Cbz-protected (S)-tyrosine in a 26-step process with an overall yield of about 1.6% [107]. The synthesis starts with obtaining alcohol (**30**) from Cbz-protected (S)-tyrosine following a previously established process [108] comprising 6 steps running in 50% overall yield and allowing the alcohol to be obtained at the decagram scale (Scheme 11). The synthesis also includes the preparation of benzo [1,3]dioxole (**31**) which was formed via remote light-mediated C-H bond activation from the corresponding quinone [107]. The preparation of the hexacyclic intermediate (**32**) on a large scale (>10 g) enabled the gram-scale synthesis of ET-743 (**29**), solving the long-standing problem of supplying this complex antitumour drug [107].

**Scheme 12:**
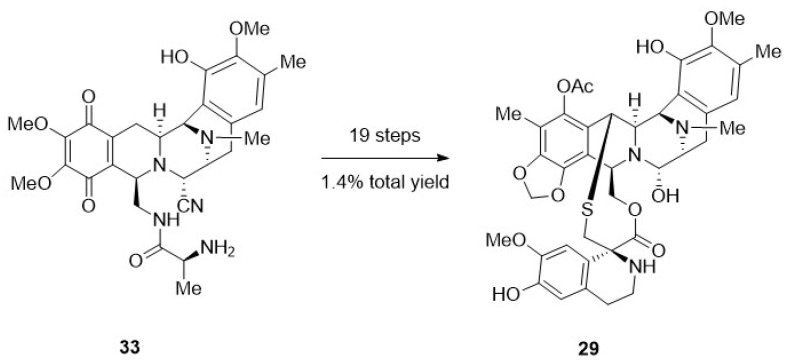
Semisynthesis of Trabectedin, ET-743 (**29**) from Cyanosafracin B (**33**) [101,109].

However, ET-743 (**29**) has been obtained by a short and straightforward semisynthetic process developed and patented by PharmaMar, starting from Cyanosafracin B, (**33**), produced by the fermentation of the bacterium *Pseudomonas fluorescens* [101,109]. Starting from Cyanosafracin B (**33**), ET-743 (**29**) was obtained on a multigram scale in a very short and direct route (19 steps only), in an economically cost-effective process with a 1.4% overall yield (Scheme 12). The chemical methodology developed in this semisynthesis has shown to be of great versatility accessing the other ecteinascidins through the N-demethylation of the fully protected intermediate leading to Ecteinascidin 729, ET-729, whose bridgehead amine allows nitrogen derivatives of the ecteinascidins and facilitates access to compounds difficult to obtain from the natural source.

In view of the total syntheses described for the different MNPs, we can say that the synthetic routes, in general, involve a large number of steps and low overall yields, obtaining few MNPs per large-scale total synthesis. Only in the case of simple compounds, such as nucleoside derivatives, the syntheses are less complicated and use a smaller number of steps, obtaining a higher number of such MNPs compared to the total number of MNPs synthesized. Notably, the semisynthesis of Plitidepsin (**21**) and Trabectedin (**29**), in one and nineteen synthetic steps, respectively, are exceptional examples of the symbiosis between both MNPs chemical and biological production methods. However, in many cases, the only way to supply the quantities of MNP needed for clinical trials leading to FDA approval and commercialization is, for the time being, solely and exclusively from their total chemical synthesis.

## 4. Chemoenzymatic Synthesis of Marine Natural Products

Chemical and enzymatic syntheses have evolved as two fundamentally distinct methodologies for the total synthesis of structurally complex natural products [110,111]. However, these approaches exhibit complementary strengths and limitations in the production of valuable molecular entities. The integration of both strategies can potentially overcome individual shortcomings and enhance overall synthetic efficiency in accessing intricate natural scaffolds [112,113].

Biological processes involve fewer reaction steps, high specificity, and enantioselectivity and can be therefore preferred over chemical processes as happens in the synthesis of 2-keto-3-deoxy-galactonate, a precursor for various applications in food, pharmaceuticals, and other industries, from marine biomass via a biocatalytic enzyme-mediated process [114]. The process begins with the depolymerization of agarose, the most abundant polymer in red macroalgae biomass, using endo-type agarases (Aga16B from *Saccharophagus degradans*, NAOH, and SdNABH from *Saccharophagus degradans 2*–*40T*) which break down the polymer into smaller oligosaccharides, including 3,6-anhydro-*L*-galactose (AHG) which is converted to 3,6-anhydro-*L*-galactonate by AHG dehydrogenase (VejAHGD). Following this, 3,6-anhydrogalactonate is isomerized to 2-keto-3-deoxygalactonate by the enzyme 3,6-anhydrogalactonate cycloisomerase (VejACI). These two last enzymes are obtained from *Vibrio* sp. EJY3 [114].

Another example of the use of enzymes in sustainable production of high-value products is the production of chitin and chitosan from shrimp byproducts. Chitosan demonstrates significant potential in the nanotechnology sector for the development of advanced drug delivery systems, particularly in the context of targeted anticancer therapeutics [115]. The conversion of cephalothorax from *Penaeus vannamei* into chitin involves a series of enzymatic and chemical treatments. Initially, chitin is extracted through a combination of enzymatic proteolysis, acid demineralization, and alkaline hydrolysis. This process yields chitin with a high degree of purity, characterized by a 96% acetylation degree and 88% crystallinity, while maintaining low levels of impurities such as ashes, lipids, and proteins with a yield of 30%. Chitin can subsequently be deacetylated to produce chitosan, a cationic derivative with enhanced functional properties. Effluents generated during the process, rich in proteins and pigments like Astaxanthin, can be recovered for further applications, such as producing fish protein hydrolysates. Thanks to this dual approach the recovery of valuable biopolymers is maximized, and waste is valorized, making the process more environmentally friendly [116].

Over the past 20 years, combined synthetic–enzymatic systems have enabled multiple total synthesis endeavors, and the use of enzymes is becoming routine in some industrial process [117]. So, the synthesis of therapeutics through the synergistic combination of chemical and enzymatic transformations has emerged as a promising area of research, allowing for the harnessing of the exceptional chemo-, enantio- and stereoselectivity of enzymatic transformations under mild conditions while utilizing various organic reactions to create chemical bonds that may not be achievable through enzymatic methods alone [4,118,119].

Despite this potential, comprehensive investigations into synergistic approaches combining both chemical synthesis and biocatalysis for the total synthesis of structurally intricate natural products remains underexplored with the exception of semi-synthetic methodologies [120,121], as was commented in the previous section.

Few reviews have been published on the synthesis of marine natural products via enzymatic reactions [122,123,124,125]. The scope of enzymatic transformations is limited and designing novel non-natural reactions using biological systems remains a challenge.

This section highlights the potential of engineered enzymes in the synthesis of complex natural products and the creation of biologically relevant compounds, demonstrating how the integration of chemical and enzymatic synthesis provides a powerful framework for the total synthesis of complex natural products. This approach is more efficient and sustainable compared to conventional methods

For example, chemoenzymatic strategies have been extensively used for synthesizing complex glycans because glycosyltransferase (GT)-mediated glycosylation offers excellent regio- and stereoselective control of glycan production without using protecting groups [126]. The application of this methodology has been particularly valuable in the production of natural marine gangliosides, which have attracted considerable attention owing to their complexity and neurotrophic activity [127,128].

**Figure 3:**
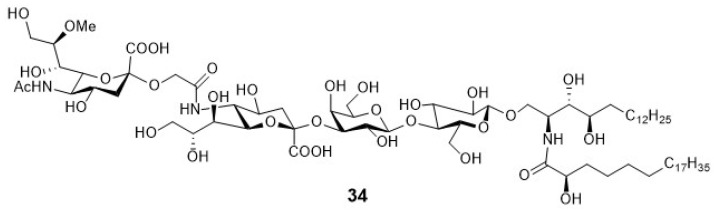
Predominant structural isomer of LLG-3 (**34**) from *Linckia laevigata*.

**Figure 4:**
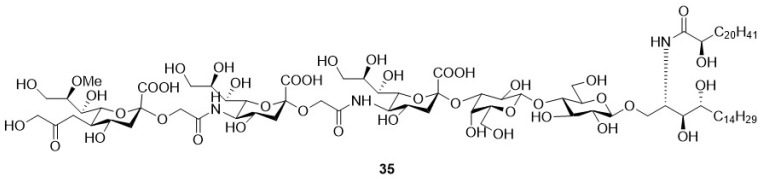
Structure of ganglioside LLG-5 (**35**).

The use of an engineered endoglycoceramidase II (EGCase) from *Rhodococcus* sp. which has been modified to catalyze the synthesis of glycosphingolipids rather than their degradation facilitated the coupling of oligosaccharides with various sphingosines allowing for the creation of structurally diverse gangliosides [129], like LLG-3 (**34**) (Figure 3) with a total yield around an 18–30% through 9 steps. Also, the ganglioside LLG-5 (**35**) (Figure 4) which has an important neuritogenic activity was chemoenzymatically prepared thanks to the stereoselectivity of sialyltransferase-catalyzed α (2,3)-glycosylation in 10 steps with a total yield of 12%, starting from a known per-*O*-benzoyl lactosyl trichloroacetamide donor [127].

Another representative example of a green synthetic strategy is the synthesis of (S)-norcoclaurine, a benzylisoquinoline alkaloid with significant pharmacological activities and biological effects, such as adrenergic β-agonist, anti-inflammatory, cardiotonic, and fibrinolytic properties [130]. Due to its limited availability from marine and terrestrial sources, a sustainable synthetic approach was developed. The process begins with the oxidative decarboxylation of tyrosine to generate 4-hydroxyphenylacetaldehyde, followed by the addition of dopamine and the recombinant enzyme (S)-norcoclaurine synthase (NCS), along with ascorbate to prevent oxidation. The enzyme facilitates a Pictet–Spengler condensation reaction, achieving (S)-norcoclaurine through a one-pot, two-step process with a yield exceeding 80% and an enantiomeric excess (ee) of 93% [131].

### 4.1. Enzymatic Desymmetrization of Meso-Compounds, Kinetic Resolution, and Deracemization

The application of biocatalysts for the desymmetrization of meso-compounds, kinetic resolution, and deracemization of racemic marine-derived alkaloid natural products has received significant attention to afford enantiomerically pure compounds [124]. For example, the synthesis of Paecilocin A, a compound which exhibits a promising inhibitory activity against pathogenic bacteria [132], involves the elegant use of lipase-mediated kinetic resolution of the propargyl alcohol, undec-1-yn-3-ol, to create the stereocenter of the target molecule (97% ee and yield of 46%), alongside the Alder–Rickert reaction to produce the aromatic core. Starting from the commercially available nonanal and thanks to the Novoenzyme 435 lipase, the compound was synthetized through nine steps and with a yield around 11% [133].

The alkaloid (−)-Gephyrotoxin-223, a 3,5-disubstituted indolizidine with interesting neurological activities, was formally synthesized from pyridine-2,6-dicarboxylic acid. Its chemoenzymatic total synthesis involved a desymmetrization step catalyzed by lipase. *Aspergillus niger* lipase was employed in the enzymatic hydrolysis of *N*-carbobenzoxy-*cis*-2,6-diacetoxy-methylpiperidine (5 days, 83% yield, ee ≥ 98%), while *Candida antarctica* lipase catalyzed the acetylation reaction of *N*-carbobenzoxy-*cis*-2,6-dihydroxy-methylpiperidine in only 3 h, good yield (80%) and high enantiomeric purity (ee ≥ 95%) [134].

Large quantities of enantiomerically enriched starting material ((*S*)-5-ethoxy-3-hydroxy-5-oxo pentanoic acid), which is crucial for subsequent steps in the synthesis of the C1–C11 fragment of (+) Peloruside A, a macrocyclic secondary metabolite with potential activity against cancer, neurodegeneration and autoimmune disease [135], were obtained thanks to the enzymatic desymmetrization of diethyl 3-hydroxyglutarate, catalyzed by immobilized lipase B from *Candida antarctica* (CAL B) [136]. The reaction occurred efficiently within 45 min at room temperature and pH 7, yielding the product with high enantiomeric purity and 95% efficiency, up to 50 g scale. The synthetic strategy included a Sharpless dihydroxylation and substrate-controlled aldol coupling to establish chiral centers at specific positions, ultimately achieving the desired stereochemistry for the aldehyde fragment. The overall yield for this synthesis was 28% over 15 steps, demonstrating the method’s efficiency and scalability for producing significant quantities of the target compound [136].

Cyptophycins are a family of macrocyclic depsipeptide natural products that display exceptionally potent antiproliferative activity against drug-resistant cancers [137]. Sherman et al. demonstrated the unique, inherent flexibility of both cryptophycin thioesterase (CrpTE) and cryptophycin epoxidase (CrpE), a versatile set of enzymes that catalyze macrocyclization and epoxidation, for production of a new set of unit A variants of these potent tubulin-binding compounds, and their potential as versatile biocatalysts to provide both native and non-native cryptophycin analogs [138,139]. The conversion of the cyclization step which generated the 16-membered depsipeptide ring with CrpTE was in the range of 67–96%, while maximum epoxidation reaction conversion with CrpE was 39% [139].

**Scheme 13:**
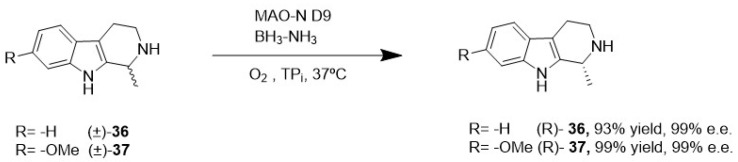
Enzymatic deracemization of racemic Eleagnine (**36**) and Leptaflorine (**37**) via a monoamine oxidase (MAO-N)-mediated oxidation-reduction.

The use of oxidoreductases is not as common as the use of lipases for desymmetrization of meso compounds or resolutions. This is likely because oxidoreductases require cofactors, and therefore working with isolated enzymes would be prohibitively expensive, unless recycling loops are introduced into the experimental protocol. However, we can find different examples as the study of Ghislieri et al. that highlights the effectiveness of engineered monoamine oxidase from *Aspergillus niger* variants (MAO-N) in deracemization reactions of marine-derived alkaloids (±)-Eleagnine (**36**), a potent analgesic, anti-inflammatory and weak antioxidant [140] and (±)-Leptaflorine (**37**), a known psychedelic [141] into their enantiomerically pure forms (Scheme 13). The process involved repeated cycles of selective oxidation on (*S*) enantiomer followed by non-selective reduction in the resulting imine using BH_3_-NH_3_. The strategy yielded (*R*)-Eleagnine (**36**) with a 93% yield and (*R*)-Leptaflorine (**37)** with a 99% yield, both maintaining 99% ee [142].

### 4.2. Enzymatic Carbon–Carbon Bond Formation

Thanks to cyclohexylamine oxidases and flavin-dependent amine oxidases, it is possible to achieve carbon–carbon bond formation, a key reaction in organic synthesis to construct the framework of organic molecules.

To the best of our knowledge, Psilocybin, a tryptamine alkaloid of significant interest due to its potential therapeutic applications in treating end-of-life anxiety and therapy-refractory depression [143], has not been isolated from marine organisms. Nevertheless, the synthesis of 6-methylated psilocybin is discussed in this article as an example of how enzymatic processes can be utilized as an efficient and sustainable strategy for gram-scale production [144]. Fricke et al. described a sequential enzymatic synthesis of 6-methylated Psilocybin that began with 4-hydroxy-6-methylindole as the starting material, which was converted to 4-hydroxy-6-methyl-L-tryptophan using the recombinant enzyme tryptophan synthase (TrpB) from *Psilocybe cubensis*. This compound then served as a co-substrate in a one-pot reaction involving three additional recombinant enzymes: PsiM for N-methylation, PsiD for decarboxylation, and PsiK for phosphorylation. This sequential enzymatic process resulted in the formation of 6-methylated Psilocybin with a yield of 75% across two enzymatic steps. This approach not only enhances the production of Psilocybin but also paves the way for exploring other analogs and derivatives with potentially improved therapeutic properties [144].

The therapeutic potential of tetrahydroisoquinoline (THIQ) alkaloids, potent antitumor agents derived from tyrosine such as saframycins, jorunnamycins, Ecteinascidin 743, or Lurbinectedin, which replaces the THIQ unit with a spiro-fused β-tetrahydrocarboline moiety, coupled with the increasing demand for their artificial production has made the common THIQ scaffold a significant target for both chemical synthesis and engineered biosynthesis [145,146,147].

**Scheme 14:**
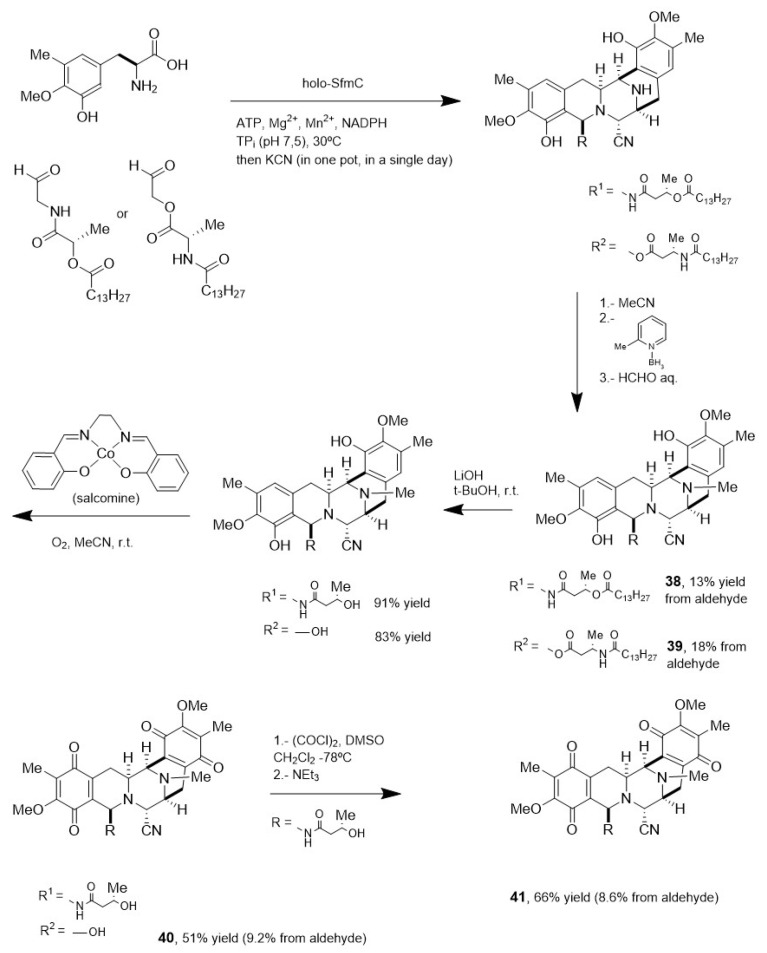
Details of the chemo-enzymatic synthetic process and total synthesis of Jorunnamycin A (**40**) and Saframycin A (**41**).

A recent collaboration between the Oikawa and Oguri groups achieved the shortest known route, integrating chemical and enzymatic methods, to efficiently synthesize Jorunnamycin A (**40**) and Saframycin A (**41**), two bis-tetrahydroisoquinoline (THIQ) alkaloids [148]. The key step involved the employment of phosphopantetheinylated SfmC enzyme which possesses unique catalytic functions. This non-ribosomal peptide synthase assembled two molecules of a tyrosine derivative and a peptidyl aldehyde bearing a long fatty acyl chain to construct the bis-THIQ scaffold, utilizing iterative Pictet–Spengler reactions to install stereogenic sp^3^ carbon centers, instead of typical amide bond formation. The implementation of sequential chemical manipulations, involving cyanation at C-21 and *N*-methylation using formaldehyde and 2-picoline borane yielded 18% and 13% of the intermediates **39** and **38**, which were further processed to produce Jorunnamycin A (**40**) and Saframycin A (**41**) through a 4-pot or 5-pot process (Scheme 14), respectively [149].

This chemo-enzymatic approach, which does not require the isolation of any intermediates, greatly reduces the time and labor involved in assembling the highly elaborated pentacyclic THIQ scaffold, constituting a versatile platform for the structural diversification of bis-THIQ alkaloids. The introduction in the synthesis of this sequential cyanation and *N*-methylation has been proven vital for enhancing the scalability and reproducibility of the chemo-enzymatic process, enabling the synthesis of pentacyclic products in a single day through a two-pot process.

**Scheme 15:**
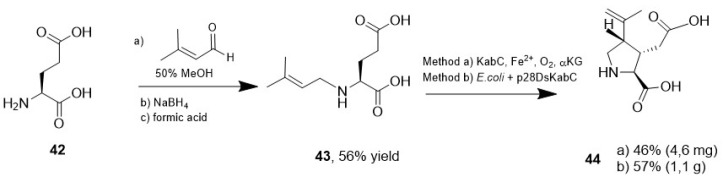
Chemoenzymatic synthesis method (**a**) or biotransformation with *E.coli* cells expressing dskabC gene in a pET28 vector method (**b**) of kainic acid (**44**).

Another example of an enzymatic C-C bond formation is the synthesis of kainic acid (**44**), an anthelmintic agent to treat parasitic worm infections [150]. Despite being a monocyclic compound (trisubstituted pyrrolidine ring), it presents a synthetic challenge because of its three contiguous stereocenters. Although there are already many scalable synthetic processes for **44**, these procedures require at least six synthetic transformations with yields of less than 40% [151]. Moore et al. [152] introduced a concise chemoenzymatic synthesis of **44** using a α-ketoglutarate-dependent dioxygenase-induced oxidative cyclization (Scheme 15). This process involved a reductive amination of *L*-glutamic acid (**42**) with 3-methylcrotonaldehyde to synthesize prekainic acid (**43**), which was then converted to kainic acid (**44)** by a homolog of an α-ketoglutarate-dependent dioxygenase (DsKabC) that catalyzed the stereocontrolled formation of the trisubstituted pyrrolidine ring with a 46% yield on a small scale (10 mg).

To improve scalability, bypassing the need to purify the enzyme, **43** was directly convert to **44** in *Escherichia coli* cells expressing DsKabC with a 57% yield on a gram scale, representing a significant improvement over previous synthetic methods that required at least six steps.

### 4.3. Biocatalytic Oxidation

One example of a biocatalytic heteroatom oxygenation is the use of OxaD, an indolic nitrone synthase isolated from the marine-derived fungus *Penicillium oxalicum* F30 in the synthesis process of indolic nitrones with potent bioactivities [153]. This flavin-dependent oxidase transformed, in a single biocatalytic-step, Roquefortine C and its semisynthetic derivatives into their corresponding nitrone functionalized indolines isolated in pure form with a satisfactory yield (greater than 20%) as well as (+)-Stephacidin A into Avrainvillamide, a naturally occurring alkaloid with antiproliferative effects [154], with a total conversion of 20% [153].

The practical utility of biocatalytic C–H hydroxylation was demonstrated in the concise and efficient syntheses of Manzacidin C, a rare bromopyrrole alkaloid that contains a unique tetrahydropyrimidine motif and densely substituted amino acid derivatives [155], developed by Zwick and Renate [156]. By exploiting the substrate promiscuity of Fe/αKG leucine 5-hydroxylase (GriE), azidoleucine was selectively hydroxylated at the δ position in >95% conversion on 130 mg scale [156].

**Scheme 16:**
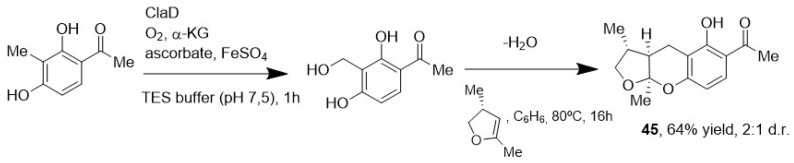
One pot chemoenzymatic cascade for the total synthesis of (−)-Xyloketal D (**45**). ClaD: non-heme iron (NHI) dependent monooxygenase.

C–H hydroxylation and oxidative dearomatization are effectively combined with traditional synthetic methods to facilitate access to natural products and their analogs [157]. For instance, the non-heme iron (NHI)-dependent monooxygenase ClaD was developed as a biocatalytic platform for the benzylic hydroxylation of *ortho*-cresol compounds, enabling a direct route to ortho-quinone methides through water loss under mild conditions. This approach was utilized in the total synthesis of (−)-Xyloketal D (**45**), a compound with antioxidant activity and neuroprotective effects, achieved through a fast and efficient one-pot chemoenzymatic cascade. By trapping one of these active precursors with a chiral dienophile, the synthesis reaches a yield of 64% and a 2:1 diastereomeric ratio (Scheme 16) [158].

Another example is the synthesis of Saxitoxin, a potent neurotoxin used in neurochemical and molecular biology research and its derivative 11-β-hydroxy saxitoxin. It involves the action of specific Rieske oxygenases, SxtT and GxtA, respectively, which facilitate stereoselective hydroxylation reaction of β-saxitoxinol or Saxitoxin. Additionally, the O-sulfotransferase SxtSUL is involved in the selective sulfanation of Saxitoxin derivatives [159,160].

**Scheme 17:**
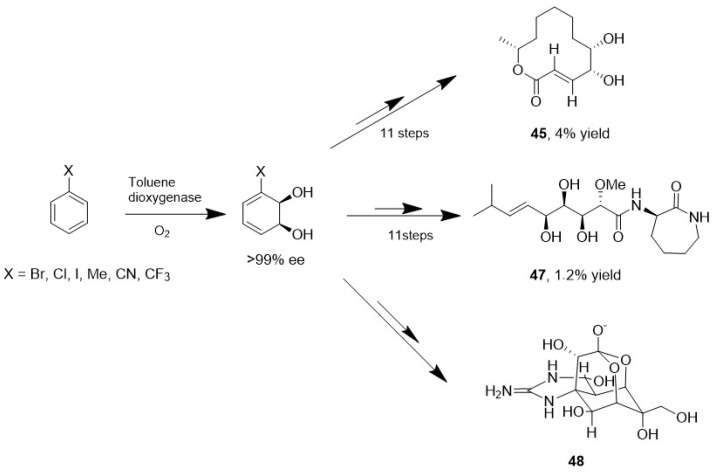
Chemoenzymatic synthetic routes to diverse compounds (Cladosposlide A (**46**), *ent*-bengamide E (**47**), (−)-Tetrodotoxin (**48**)) utilizing arene dihydroxylation as a key enzymatic step.

The microbial-mediated enzymatic *cis*-dihydroxylation of aromatic substrates facilitated by genetically engineered bacterial strains that overexpress the enzyme toluene dioxygenase represents a valuable biotransformation process for the production of chiral synthons with high optical purity, necessary to achieve the overall synthesis of different compounds (Scheme 17). The resultant chiral diols have been extensively employed as key intermediates in numerous total syntheses of complex natural products and pharmaceutically relevant compounds.

For example, chiral *cis*-1,2-dihydrocatechols are readily obtained on a large scale through a genetically engineered strain of *Escherichia coli* responsible for the microbial dihydroxylation of chlorobenzene, bromobenzene or iodobenzene since no other efficient chemical method is known [161]. This compound was used together with (*R*)-pent-4-en-2-ol in the overall synthesis of Cladospolide A (**46**) achieved in 11 steps with a 4% overall yield. The process employed ring-closing metathesis (RCM) as a pivotal step, demonstrating a high degree of convergence and efficiency. Enantiomerically pure (*R*)-pent-4-en-2-ol was accessed by a two steps resolution protocol that could be run on a multigram scale, using the lipase CALB to achieve high enantiomeric purity [162].

Furthermore, the use of methyl (6*E*)-6,7,8,9-Tetradeoxy-3,4,5-tris-*O*-[(1,1-dimethylethyl) dimethylsilyl]-8-methyl-2-*O*-methyl-l-gulonon-6-enonoate derived from *cis*-1,2-dihydrocatechol allowed the chemoenzymatic synthesis of *ent*-bengamide E (**47**), a natural product isolated from sponges of the *Jaspidae* family which exhibits significant anti-proliferative effects [163]. It was obtained with an overall yield of approximately 1.2% over 11 steps [164].

Clearly, enzymatic dihydroxylation of arenes produces a substantial reduction in complexity of the overall synthesis of (−)-Tetrodotoxin (**48**) enhancing the efficiency of the synthesis of key intermediates compared to previously reported methods of the Fukuyama, Alonso or Sato’groups: The resulting diene diols were transformed into Fukuyama’s intermediate in six steps, into Alonso’s intermediate in nine steps, and into Sato’s intermediate in ten steps [165]. (−)-Tetrodotoxin (**48**) is a natural neurotoxic alkaloid found in the ovaries and liver of many species within the family Tetraodontidae [166] and has shown potential effectiveness against neuropathic pain associated with chemotherapy-induced peripheral neuropathy [167].

Thanks to the work of various research groups, including those led by Tang [168], Narayan [158] and Watanabe [169], several FAD-dependent monooxygenases (FDMOs) that facilitate site- and stereo-selective oxidative dearomatization of highly substituted resorcinol intermediates have been characterized. These enzymes require only molecular oxygen and a nicotinamide cofactor, enabling catalysis under mild conditions while maintaining high site- and enantioselectivity. By leveraging the distinct site selectivity and stereoselectivity of various FDMOs, researchers can develop efficient synthetic pathways to access a diverse array of natural compounds, including tropolones and azaphilones.

Azaphilones constitute an extensive family of natural products isolated from fungal sources. Their relevance in the field of medicinal chemistry stems from the diversity of structural features they possess, which confers a broad spectrum of biological properties. Among the most notable activities of these compounds are antineoplastic potential [170], antiviral capacity [171] or anti-inflammatory effects [172].

Current synthetic strategies for the preparation of azaphilones, compounds characterized by an oxygenated pyranoquinone bicyclic core with a single tetrasubstituted carbon, are primarily based on either the cyclization of linear precursors [173] or more frequently the oxidative dearomatization of highly functionalized resorcinol intermediates [174].

**Scheme 18:**
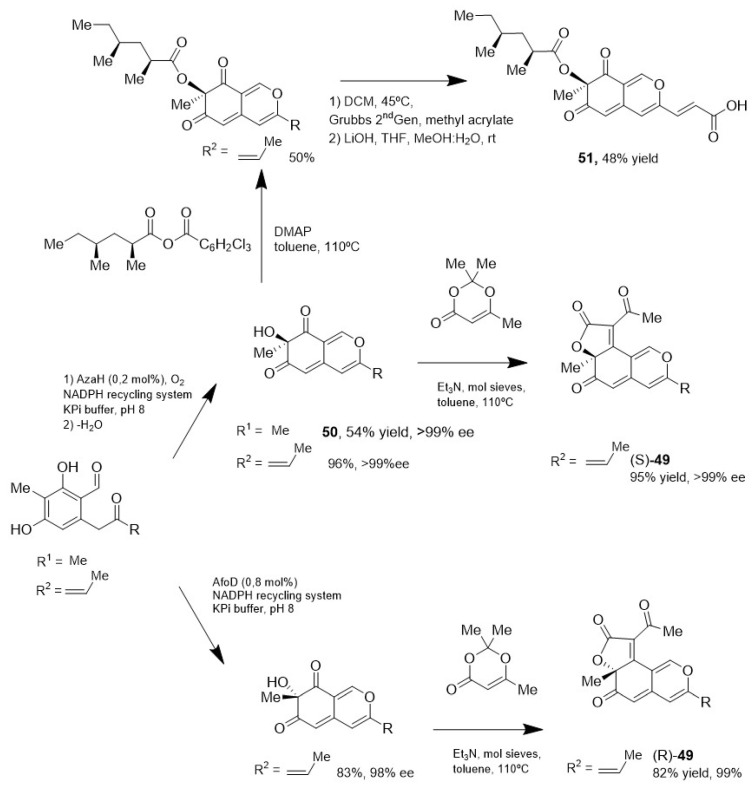
Stereodivergent, chemoenzymatic synthesis of azaphilone natural products. Trichoflectin (**49**), Azaphilone (**50**), and Lunatoic acid (**51**).

Many biologically active azaphilone natural products exhibit either R- or S-configuration at the C-7 stereocenter which differs depending on the specific compound. Several biocatalysts exhibiting complementary site- and stereoselectivity have been utilized in the stereodivergent, chemoenzymatic synthesis of azaphilone natural products [175]. FAD-dependent monooxygenases (FDMOs), AzaH and AfoD, which provide complementary stereochemical outcomes, have enabled a stereodivergent chemoenzymatic total synthesis of the angular azaphilone natural product Trichoflectin (**49**) [176]. This synthesis achieved (*S*)-**49** with a yield of 95% and an enantiomeric excess (ee) greater than 99% (with AzaH), while (*R*)-**49** was obtained with a yield of 82% and 98% ee using AfoD (Scheme 18).

Aza H mediated a site- and stereoselective oxidative dearomatization of resorcinol compounds (R_1_ = Me) producing the corresponding enol which subsequently cyclized spontaneously to yield the natural Azaphilone (**50**), isolated with a yield of 54% and an enantiomeric excess (ee) greater than 99% [176].

Additionally, Lunatoic acid (**51**) was obtained with a final yield of 48% [157], showcasing the versatility and utility of these biocatalytic strategies in accessing a range of natural products (Scheme 18).

On the other hand, the FAD-dependent monooxygenase SorbC catalyzes a C-5 hydroxylative dearomatization of Sorbicillin (**52**) derivatives, demonstrating different site selectivity compared to other monooxygenases like the mentioned AzaH and AfoD. This enzyme, derived from the Corbiculid (**52**) biosynthetic pathway, allows streamlined total syntheses accessing a broad range of highly complex members of the natural sorbicillinoid family, a large family of fungal natural products, with over a hundred isolated derivatives known for their complex structures and significant biological activities, including antibiotic and antiviral properties [177].

**Scheme 19:**
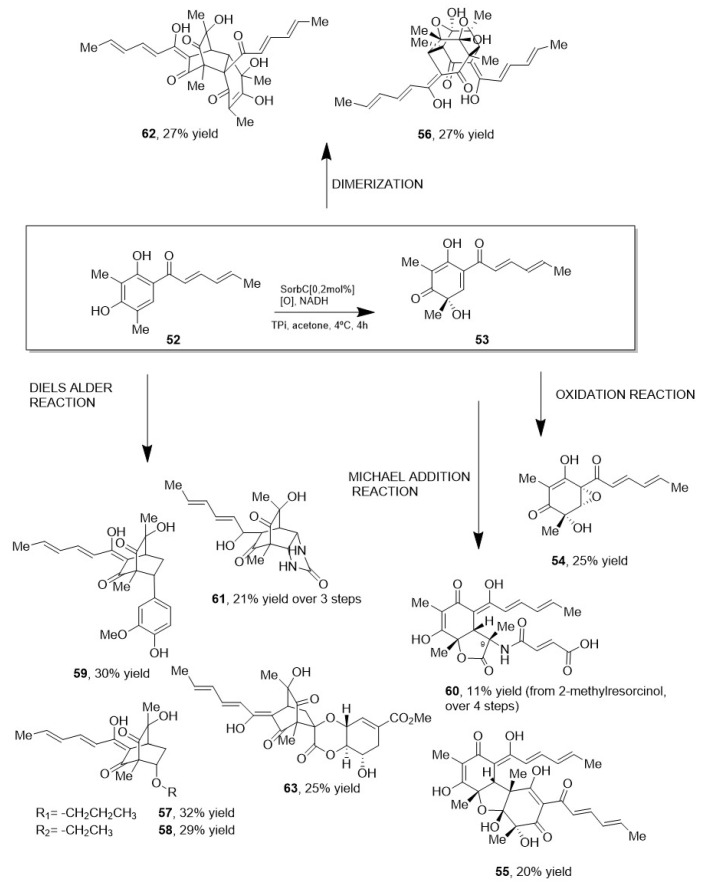
Enantioselective, one-pot chemoenzymatic routes for the total synthesis of various functionalized sorbicillinoid natural product: (+) epoxysorbicillinol (**54**), Bisvertinolone (**55**), Trichodimerol (**56**), Rezishanone B (**57**), Rezishanone C (**58**), Sorbicatechol A (**59**), Sorbicillactone A (**60**), urea sorbicillinoid (**61**), Bisorbicillinol (**62**), and Spirosorbicillinol C (**63**). Biocatalytic dearomatization step obviates the need for stoichiometric chiral reagents in the synthesis of the highly reactive Sorbicillinol (**53**) which undergoes various coupling reactions acting as both a Michael acceptor and donor, as well as an electron-rich cyclic diene and dienophile in Diels–Alder reactions.

Overall, the use of this biocatalytic oxidation strategy results in a dramatic improvement in synthesis economy over previous approaches to biomedically interesting sorbicillinoids, both in terms of step counts and reaction yields. Furthermore, the biocatalytic dearomatization step eliminates the need for stoichiometric chiral reagents in the synthesis of the highly reactive Sorbicillinol (**53**) [125] and exhibits exceptional stereocontrol achieving enantiomeric excess greater than 99.5%. Its versality in undergoing various coupling reactions—acting as both a Michael acceptor and donor, as well as an electron-rich cyclic diene and dienophile in Diels–Alder reactions—has enabled the development of enantioselective, one-pot chemoenzymatic routes for the total synthesis of various functionalized sorbicillinoid natural products (Scheme 19), including monomeric sorbicillinoids as Epoxysorbicillinol (**54**), Oxosorbicillinol or Sorrentanone, bisorbicillinoids as Bisvertinolone (**55**), Trichodimerol (**56**) and Sorbiquinol and hybrid sorbicillinoid as Rezishanone B (**57**) and C (**58**), Sorbicatechol A (**59**), and Sorbifuranone [178,179].

For example, the reaction of Sorbicillinol (**53**) with an azlactone nucleophile produced Sorbicillactone A (**60**) in just four steps, achieving an overall yield of 11% [180]. This strategy not only simplifies the synthetic route but also enables the synthesis of various C-9 alkyl Sorbicillactone analogs, expanding the structural diversity of this natural product class. Previous synthetic efforts required 12 linear steps and yielded only 0.13% of *rac*-**60** [180].

The addition of *n*-butyl vinyl ether and ethyl vinyl ether as dienophiles to Sorbicillinol (**53**) yielded Rezishanones B (**57**) and C (**58**) in a 29% yield, showcasing the efficiency of this method compared to traditional multi-step synthetic approaches. Additionally, the Weitz–Scheffer epoxidation method has enabled the stereoselective synthesis of (+)-Epoxysorbicillinol (**54**) [179].

Sorbicillinol (**53**) subjected to a bisacylated urea led to a [4 + 2] cycloaddition reaction in the total synthesis of a urea sorbicillinoid natural product. For example, the Urea sorbicillinoid (**61**) was achieved in a 21% yield over three steps [157].

A stereoselective, one-pot biocatalytic total synthesis using an enzymatic oxidative dearomatization/dimerization cascade enabled the synthesis of structurally diverse bisorbicillinoids, including those formed via Michael addition/ketalization and Diels–Alder cycloaddition [178]. The ability to rearrange Bisorbicillinol (**62**) into Bisorbibutenolide or Bisorbicillinolide further enhances the synthetic utility of this method facilitating future studies into their biological activities and therapeutic potential. Although this method is suitable for a fast preparation and with better yield than those obtained through different total chemical synthesis, target compounds and unnatural analogs are obtained in mg quantities [178].

Using the strategy employed in the synthesis of dimeric sorbicillinoids, hybrid derivatives also present in nature as spirosorbicillinoids like Spirosorbicillinol C (**63**), Saturnispol C, Trichsorbicillin A, and its demethylated analog have been obtained. They were proposed to be derived from either a Diels–Alder or a Michael reaction of monomeric sorbicillinoid diene and a second non-sorbicillinoid dienophile [181].

As mentioned before, the employment of SorbC resulted in stable oxidation products that serve as valuable intermediates. Recent advancements have showcased its broad synthetic potential which facilitated the oxidative dearomatization of different hexasubstituted phloroglucinol (Sorbicillin-type synthetic precursors) which have different substitution patterns at the aromatic ring system or highly substituted (α-acylated or -formylated) phenols [182]. Also, when a diverse set of dienophiles was employed to quench the reaction, the structural diversification of the bicyclo [2.2.2] octane core by varying the natural 2-methoxyphenol substituent was allowed. This flexible biocatalytic approach facilitated the rapid generation of milligram quantities of a diverse library of sorbicillinoid Diels–Alder analogs which is essential for structure–activity relationship studies. Yields ranged from 21% to 32%, showcasing the efficiency of the chemoenzymatic strategy [183].

**Scheme 20:**
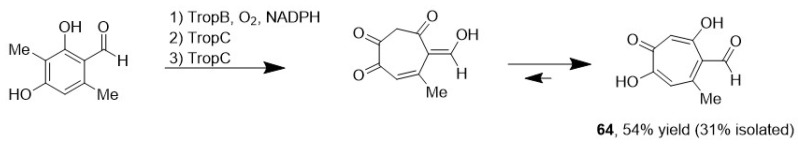
Synthesis of the tropolone natural product, Stipitatic aldehyde (**64**), through a cascade of three enzymatic reactions.

Finally, as we can observe in Scheme 20, a cascade of three enzymatic reactions yielded the tropolone natural product Stipitatic aldehyde (**64**). TropB facilitated the hydroxylative dearomatization of resorcinol to produce an *o*-quinol product which could then be further processed by the NHI monooxygenase (β-ketoglutarate dependent non heme iron enzyme) TropC to yield corresponding 1,2-diol which readily underwent ring expansion in the presence of TropC to afford a seven-membered ring, whose tautomerization afforded the stipitatic aldehyde. Biotransformations with whole *Escherichia coli* cells containing heterologously expressed protein gave a comparable conversion on a gram scale to that observed on analytical scales. Within 2 h, a complete conversion of Stipitatic acid (**64**) had been achieved [157].

These are some representative examples where the use of biocatalysis in conjunction with synthetic chemistry presents a promising strategy for the efficient production of therapeutics, particularly in the context of marine natural products.

The recent explosion in genomic data availability, advances in bioinformatics, analytical techniques, and enzyme engineering, together with the growing understanding of biocatalytic processes, will increase the potential of biocatalysis to meet the demands of modern pharmaceutical manufacturing. Together, these advances will provide more opportunities to develop new enzymatic transformations and strategically integrate them into multi-step syntheses. Overall, the application of enzyme-catalyzed cascades not only streamlines synthetic routes but also it is aligned with the principles of green chemistry, offering a sustainable alternative to traditional chemical synthesis methods.

However, yields for marine-derived natural products production via (chemo)enzymatic approaches have not generally exceeded a few milligrams per liter, quantities that allow their biochemical function characterization but are insufficient to ensure a (pre)clinical supply. The challenges associated with preparative-scale biocatalytic reactions can serve as a barrier to enzymes being embraced as tools for synthesis. Complications with enzyme supply, labor-intensive protein purification, substrate solubility in aqueous buffer, and cofactor expense can all be hurdles to preparative-scale biocatalysis.

## 5. Production of MNPs Using Biotechnological Approaches

As has been seen in the second section, obtaining drugs with a very complex chemical structure is becoming more and more feasible thanks to the continuous advances in chemical synthesis. This leads us to believe that this will be the prevailing strategy in the production of future drugs obtained from natural marine products at an industrial scale. However, given the obvious environmental problems, the high production cost of these methods and the low yields obtained, biotechnological methods offer an alternative to be considered. In this section, we summarize some of the methods currently in use, as well as some of those under development.

### 5.1. Fermentation: Axenic Macroscale Culture and Mixed Fermentation

Fermentation represents a potential solution to the supply issue. It uses microorganisms to obtain value-added products. Unlike macroorganisms, microorganisms have important advantages in terms of their adaptability and feasibility as well as in the cost of their industrial-scale fermentation. In the case of MNPs from bacterial, fungal, or microalgal sources, laboratory scale cultures are preferred over chemical processes as they involve fewer reaction steps, high specificity and enantioselectivity. If we want to obtain an MNP from an invertebrate source, this strategy is much more complex to consider because secondary metabolites usually are accumulated in only very small quantities on these organisms in nature [20].

Although natural products represent a significant number of the molecules available on the market, the pharmaceutical industry seemed to have favored synthetic molecules over natural products. However, with the discovery of silent gene clusters and the significant improvement in screening, analytical and molecular biology techniques, a renewed interest in natural products research has emerged.

New strategies are needed to increase the availability of microbial biomass and to minimize the environmental impact of organism collections. Also, it is necessary to adopt new strategies to develop sustainable in vitro approaches to culture whole organisms or individual cell types of specific marine species [184,185,186,187,188]. It is particularly important for sponges and corals, which harbor intracellular endosymbiotic microorganisms. Not only do they harbor endosymbionts, for sponges in particular it is estimated that up to 40% of the “sponge” biomass is bacterial and that the vast majority of sponge biosynthetic potential is due to these symbionts. Proper culture settings should provide optimal conditions for holobionts [184].

Culturable marine microorganisms are predicted to be a valuable source of innovative and efficient drugs [29] due to their tremendous genetic and biochemical diversity [35]. For example, actinomyces provide more than 70% of naturally occurring antibiotics [12]. Despite most marine microbes being hard to cultivate in the laboratory, some strains have already produced significant bioactive compounds that are on the market or in clinical trials. As standard conditions do not always favor fungal or bacterial production of interesting secondary metabolites [189] as compared to the constitutive ones typically produced under any conditions, one of the driving forces to produce these specific metabolites is the simulation of the native environment of marine microbes, including interactions with other microorganisms, as is often the case in nature [189]. For example, Salinosporamide A (Marizomib^®^) production, for the ongoing clinical trials, relies on large scale saline fermentation. Since under laboratory conditions microbial cultures may present a metabolomic profile different from that of their original environment, it is very important to take into account the chemical–ecological relationships that occur in their communities [190]. Sometimes, it is necessary to coculture with two or more strains to obtain the effects that a single strain could not make. The reason falls in the fact that many important biochemical pathways in microorganisms could not be finished or could be finished weakly by an individual strain, and it needs the additional stress of a competitor to upregulate.

On the other hand, marine microbes (actinobacteria, cyanobacteria, etc.) that live in symbiosis with the larger host organisms or marine associative algae [191] produce highly biologically active substances that inhibit the growth of pathogenic bacteria and viruses that would affect the vital processes of host organisms.

#### 5.1.1. Axenic Macroscale Cultures

Axenic macroscale culture in a bioreactor could represent the most efficient production strategy to reach industrial scale production of some marine drugs. It has proven useful for the industrial scale supply of many different antibacterials (Erythromycin, Vancomycin, Streptomycin, penicillins, and cephalosporins), antifungals (Griseofulvin and Amphotericin B), antiparasitics (Avermectins and Fumagillin), and immunosuppressants (Tacrolimus, Cyclosporine, and Mycophenolic acid) natural products from a wide range of terrestrial bacterial and fungal sources [192].

An important example is Turbinmicin, identified by Zhou and colleagues; it is a highly oxidized type II polyketide from the marine actinomycete *Micromonospora* sp. Turbinmicin exhibits antifungal activity by inhibiting the vesicle-mediated trafficking pathway of many potentially deadly fungi [193,194,195]. This promising new marine antifungal drug demonstrated efficacy in mouse models against multidrug-resistant yeasts and molds. In some cases, this biosynthetic method may be complemented by some semisynthetic steps to complete the scaffold of some microbial secondary metabolites; for example, the anticancer drugs Trabectedin (**29**) (Yondelis^®^) and Lurbinectedin (Zepzelca™) are semisynthetic derivatives of Cyanosafracin B (**33**) produced by axenic cultures of *P. fluorescens* [101] as previously described in Section 3, and these semisyntheses allows their industrial scale production.

**Figure 5:**
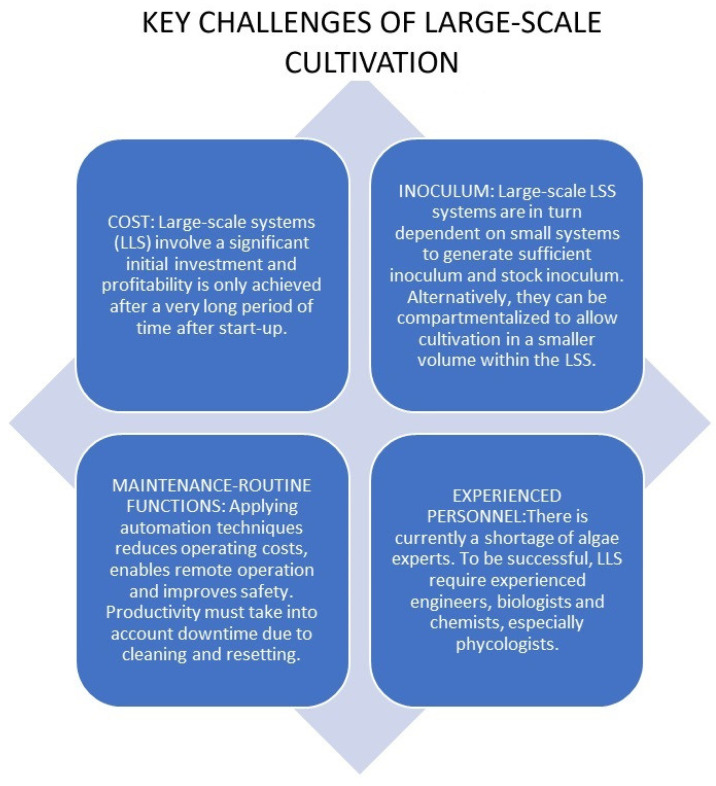
Frequently challenges in large-scale cultivation systems.

In an interesting review, Novoveská et al. [196] analyze and summarize fundamental aspects of large-scale cultivation of photosynthetic microalgae and cyanobacteria, such as culture conditions, species selection, types of large-scale culture such as open ponds, floating offshore closed photobioreactors and biofilms, water and nutrient sources, temperature, light and mixing control, among others. They also provide practical recommendations and discuss the challenges facing cost-effective large-scale systems related to economic design, efficient operation and maintenance, automation and the shortage of experienced psychologists as shown in (Figure 5).

However, many obstacles must be overcome before the sustainable production of pharmaceuticals from invertebrate-derived microbes transforms into a conventional technology as happened with Bryostatin’s synthesis [31].

Due to their cellular mechanisms that make them easily adaptable to changes in the environment and to their fast and easy growth, cyanobacteria are of interest to numerous researchers due to their potential in various areas of both nutraceutical and pharmaceutical industries. One product extracted from a cyanobacteria culture is the Spirulina, a known potent dietary supplement, that possesses antioxidant, anti-inflammatory, anticancer, and low-density lipopolysaccharide (LDL) and triglyceride-lowering effects [197].

Another example is the potent cytostatic agent Phorboxazole A that inhibits the S-phase cell cycle at subnanomolar concentrations [198], or the co-produced Phorbasides [199]. Phorboxazole A was originally extracted from a Western Australian sponge, *Phorbas* spp. These compounds can be obtained by cyanobacteria living within the host or expressed from microbial genes that have been integrated into the host sponge.

With respect to the scale-up for the industrial production of metabolites from marine-derived fungi, it can often be achieved with large-scale fermentation processes [1]. For example, the fermentation process using filamentous fungi represents the major way of Lovastatin production. Lovastatin has widespread applications, like the treatment of hypercholesterolemia and as a competitive inhibitor of HMG-CoA reductase due to multiple pharmacological effects. However, its current productivity by fungal fermentation is limited and needs to be improved. Guangshan Yao et al. (2024) [200] conclude in their recent study that the combination of strain screening and genetic engineering represents a powerful tool for improving the productivity of fungal secondary metabolites. These strategies were adopted for Lovastatin large-scale production in marine-derived *Aspergillus terreus*.

**Figure 6:**
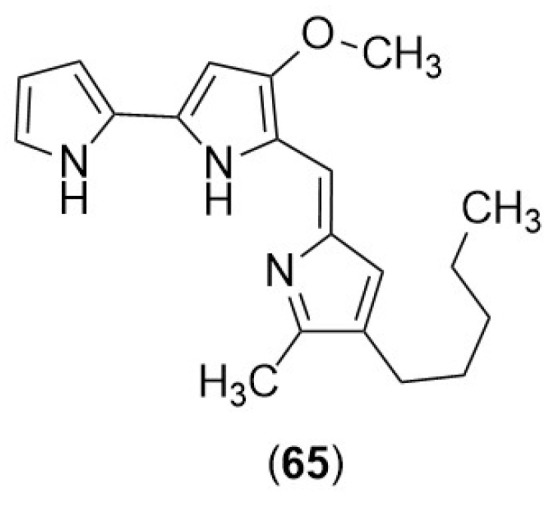
Prodigiosin (**65**) chemical structure.

**Figure 7:**
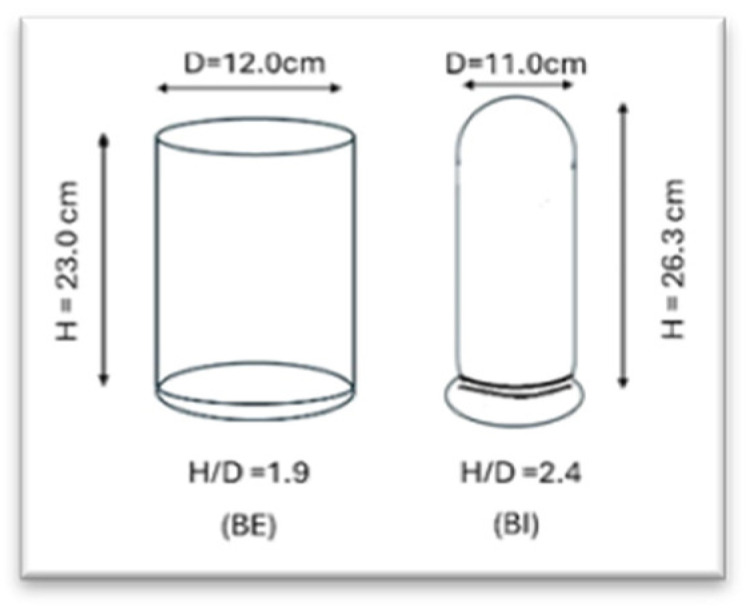
Vessel geometry: Fermac 360 (BE) and Minifors (BI).

In their work, Pereira et al. [201] developed the bottom-up process for Prodigiosin (**65**) production using a marine *Serratia rubidaea* isolated from a sample collected near a shallow water hydrothermal vent. Prodigiosin (**65**) (Figure 6) is a red bacterial pigment derived from bacterial secondary metabolites of the prodiginine family with great potential as a natural dye and drug precursor, as well as exhibiting several pharmacological properties, including antimicrobial and anticancer activities [202,203,204]. Its commercialization for biomedical applications is still scarce. Some of the main limitations are the lack of efficient bioprocesses and scaling up from laboratory to production. The product yield per biomass was found to be influenced by the concentration of cells in the inoculum. Additionally, bioreactor design was scaled up to 2 L stirred tank reactors with two different vessel geometries, Fermac 360 (BE) and Minifors (BI) (Figure 7). It was concluded that the inoculum should have a maximum concentration of 0.04 g/L DCW (Dry Cell Weight) [205] in order to obtain a considerable reduction on the extension of the lag phase, allowing for a higher amount of Prodigiosin (**65**) after 24 h. Comparison between the two 2 L bioreactor models used showed that vessel geometry contributes greatly to the fermentation outcome. Vessel geometry and a cascade control mode to regulate the dissolved oxygen concentration were shown to influence the volumetric oxygen mass transfer coefficient (k_L_a) and thus Prodigiosin production. To improve production performance, strategies were tested to mimic the aeration conditions found at the sampling site.

The geometry can limit oxygen transfer, especially at agitation speeds above 400 rpm at which the oxygen transfer observed in the tested bioreactors resulted in high kLa differences and influenced the strategy used to increase product yield.

Elicitation is a process that induces gene expression of enzymes involved in the production of secondary metabolites in plants [206]. Elicitors are classified as biotic or abiotic and are known to enhance plant defense responses against pathogens, e.g., through the production or release of toxic phytoalexins [207]. While the role of elicitors in terrestrial plants and animals is well known, little is known about the effect of biotic and abiotic elicitors on marine organisms, especially on corals [208,209]. Elicitation represents a promising tool to improve the production of metabolites that have not yet been investigated in soft corals. In cell cultures, genes encoding enzymes that catalyze biosynthetic reactions of natural products are often repressed, so a chemical signal or switch is needed to initiate the production of specific metabolites, their release if they are stored, transport, and activation [210].

In corals and their symbiotic algae, salicylic acid (SA) and its derivatives can induce the production of diterpenes and sterols. The effect of elicitation of SA and six analogs plus a systemic acquired resistance inducer on the accumulation of secondary metabolites in the soft coral *Sarcophyton ehrenbergi* together with symbiont zooxanthellae (algae) was studied by Farag et al. [206]. In addition, they investigated whether the induction effect of SA could be extended to the other coral species (*Sarcophyton glaucum* and *Lobophyton pauciliforum*). Structural analysis revealed an initial structure–activity relationship (SAR) in SA derivatives which appear to be important factors for the efficient induction of diterpenes and sterols. Following elicitation in the three corals and zooxanthellae, metabolites were extracted and analyzed by UHPLC-MS together with chemometric tools. Multivariate analysis of the UHPLC-MS data set revealed a clear segregation of SA, amino-SA, and acetyl-SA in the samples obtained. A 6- and 8-fold higher level of diterpenes, namely Sarcophytonolide I, Sarcophine, and a C28-sterol, was observed in the SA and amino-SA groups, respectively. After elicitation, the level of diepoxy-Cembratriene increased 1.5- and 2.4-fold in the SA 1 mM and acetyl-SA (aspirin) treatment groups, respectively. *Sarcophyton glaucum* and *Lobophyton pauciliforum* showed a two-fold increase in diepoxy-Cembratriene levels. These results suggest that SA may function as a general and somewhat selective diterpene-inducing signaling molecule in soft corals.

**Figure 8:**
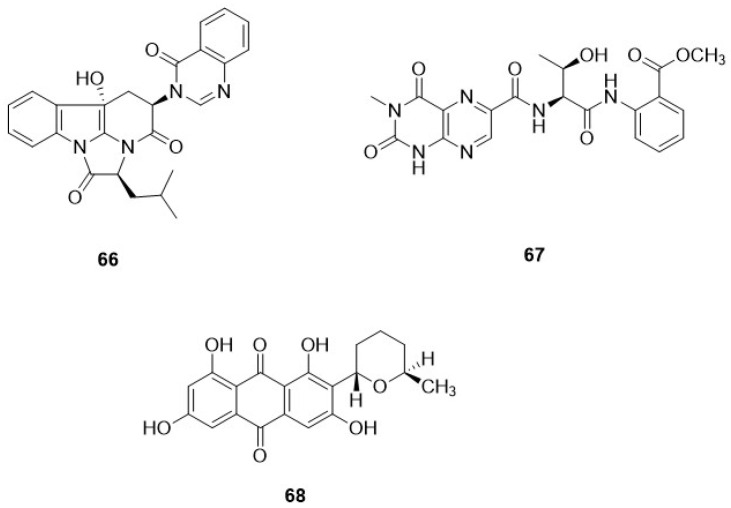
Structures of three isolated compounds from *Aspergillus carneus*: Isopropylchaetominine (**66**), Isoterrelumamide A (**67**), and 5′-epi-Averufanin (**68**).

Another fact to consider to increase MNP production by fermentation is that genomic data are not available for all microorganisms, so non-genome-dependent techniques have also been developed. One of the strategies used has been the “one strain, many compounds” (OSMAC) technique, which shows how a single strain can produce different molecules when grown under different inoculation and incubation conditions [211]. As described in a review article by Romano et al. [211], one must take into account the observed differences in metabolomic profiles between cultures on solid and liquid media. An example to illustrate the OSMAC strategy is a comparative study on changes in the metabolomic profile of a sponge-associated fungus, *Aspergillus carneus* [212]. In this study, Özkaya et al. isolated three new natural products Isopropylchaetominine (**66**), Isoterrelumamide A (**67**), and 5′-epi-Averufanin (**68**) (Figure 8), after inoculating the fungus in three different media and demonstrated how the production is depending on the composition of the culture medium in which the microorganism was inoculated; the secondary metabolites detected are not always the same [212].

#### 5.1.2. Mixed Fermentation or Co-Culture

In this technique, two or more different microorganisms are inoculated together to mimic the natural habitat in which symbiotic or competitive interactions between microorganisms are simulated and/or enhanced. Natural habitats could be characterized by limited access to resources and nutrients as well as metabolite exchange between microorganisms and/or macroscopic organisms. Competition between microbes is deliberately provoked by inducing stress factors, which stimulates the activation of silent gene clusters that were not expressed under classical culture conditions [213]. As reported by Zhu and Lin, since antibiotics can be produced in nature to provide a competitive advantage [214], it is possible that the pathways responsible for the biosynthesis of certain compounds are regulated by factors elicited by one microbe and detected by another. This proposal is justified by the following facts: Burgess et al. [215] found that antibiotic production can be induced in response to microbial antagonism and Sonnenbichler et al. [216] found that the production of specific secondary metabolites can be increased up to 400-fold when strains are grown in the presence of an antagonist.

Mixed fermentation or co-culture has been used to improve enzyme production [217] and in the food industry [218]; however, the pharmaceutical industry does not widely use this technique for the discovery of new metabolites.

Co-culture is proving to be one of the most efficient ways to induce silenced pathways, mimicking the competitive microbial environment for holistic metabolite production and regulation. Co-culture has become a gold standard methodology for metabolome expansion. It requires no prior knowledge of the signaling mechanism and genome and no special equipment for culture and data interpretation. Selegato et al. [219] have conducted a thorough review of recent studies reported on co-culture to induce secondary metabolite pathways to provide insights into experimental variables compatible with high-throughput analytical procedures. They also evaluate the publications on mixed fermentation from 1978 to 2022 in relation to co-culture types, metabolic induction and interaction effects. Likewise, in a review article 2022, Edrada-Ebel et al. study secondary metabolites of marine microorganisms and how co-culture techniques can induce their production [220]. The authors classify the articles according to the type of co-inoculated microorganisms: (1) bacteria-fungi, (2) between bacteria, and (3) between fungi. Among these examples, we indicate one from each group of co-cultivation analyzed. As a representative example, for co-cultures between fungi and bacteria they describe the work of Yu, Ding, and Ma. They co-cultured the fungus *Aspergillus flavipes* with the actinomycete *Streptomyces* sp. isolated from marine coastal sediments of the Nanji Islands in China. The mixed fermentation increased the yield of production of novel cytochalasin analogs over the corresponding fungal monoculture [221]. It is interesting to note that the co-culture process could induce the production of new compounds and increase the yield of existing metabolites from both axenic microbial cultures and not only in one of the two microorganisms involved in the co-culture. One must also consider the possibility that the co-culture could also result in a loss of production of some of the major families of fungal metabolites present in their axenic culture, while none of the bacterial metabolites have antifungal effects [222].

Another example is the production of a new antibiotic called Pestalone from the mixed fermentation of a marine fungus *Pestalotia* sp. CNL-365 and a drug-resistant marine bacterium CNJ-328 described by Cueto et al. [223]. Pestalone was not detected when either strain was grown under the same conditions separately. Oh et al. [224] found that the marine bacterium CNJ-328 induced another marine fungus *Libertella* sp. to produce four new cytotoxic metabolites designated as libertellenones in mixed fermentation. These new compounds were not observed in pure cultures of either the fungus or the bacterium.

**Figure 9:**
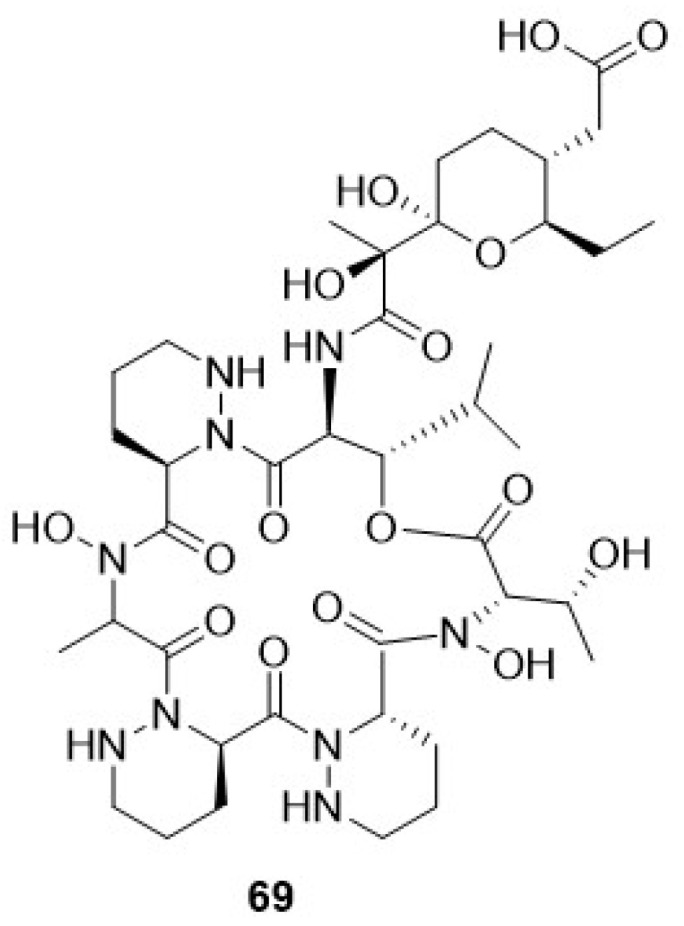
Dentigerumycin E (**69**) chemical structure.

In the second group, co-cultures between two bacterial strains, eleven publications are analyzed. Bacteria in the marine environment are found in numerous habitats such as in sediments and in association with algae and other macroorganisms. These microbes can have a free-living mode in water or sessile in the form of biofilm in which bacteria can communicate by quorum sensing [225]. A frequent problem is low biomass yields with bacterial cultures. In a co-culture environment, higher production of the target metabolites was facilitated. With higher yields, various analogs can be further evaluated for their alternative potential activities and cytotoxicity. Since fermentation parameters may change and are not directly reproducible, for industrial scale-up, it may be necessary to employ other techniques or in combination with co-culture such as hemisynthesis or metabolic engineering methods [220]. An example of how co-culture between different bacterial strains techniques allows us to obtain new bioactive molecules is the work of Shin et al., who elucidated a novel piperazine acid-bearing cyclic peptide, Dentigerumycin E, which has antimetastatic potential against breast cancer cells. It is produced by co-culture of the marine bacterium *Streptomyces* sp. JB5 and *Bacillus* sp. GN1, isolated from an intertidal mudflat in Wando, Republic of Korea [226]. *Streptomyces* sp. JB5 was co-cultured with seven different bacterial strains including *Bacillus* sp. HR1, *Paenibacillus* sp. CC2, *Brevibacillus* sp. PTH23, *Streptomyces* sp. SD53, *Streptomyces* sp. UTZ13, *Hafnia* sp. CF1, and *Mycobacterium* sp. Myc06. However, only co-culture with *Bacillus* sp. HR1, which is phylogenetically close to *Bacillus* sp. GN1, produced Dentigerumycin. While the mechanism that triggers the biosynthesis of Dentigeromycin E (**69**) (Figure 9) by *Bacillus* strains remains unclear, the results of this study with different co-culture experiments show that *Bacillus* strains, which are most closely related to *Bacillus cereus*, may share the ability to induce Dentigeromycin E (**69**) production in *Streptomyces* sp. JB5. Complete sequencing of the genome of *Streptomyces* sp. JB5 allowed the identification of the biosynthetic gene cluster (BGC) of Dentigerumycin E (**69**) and thus, confirmed that it was indeed produced by *Streptomyces* strain.

The third type of co-culture analyzed by the review article [220] is co-cultures between two fungal strains. In this section, fourteen relevant articles were selected to describe the co-culture of two fungal species. The isolation of fungal samples from the marine environment has received increasing attention as a new source of interesting metabolites despite the fact that it is more difficult to differentiate a marine fungus from a terrestrial fungus compared to bacteria. Unlike bacteria, marine fungi do not necessarily need sea salts in their culture medium to grow. So, they considered both marine and marine-derived fungi. The chemical diversity of genetically encoded small molecules in filamentous fungi may prove to be an option to consider in the discovery of new compounds.

**Figure 10:**
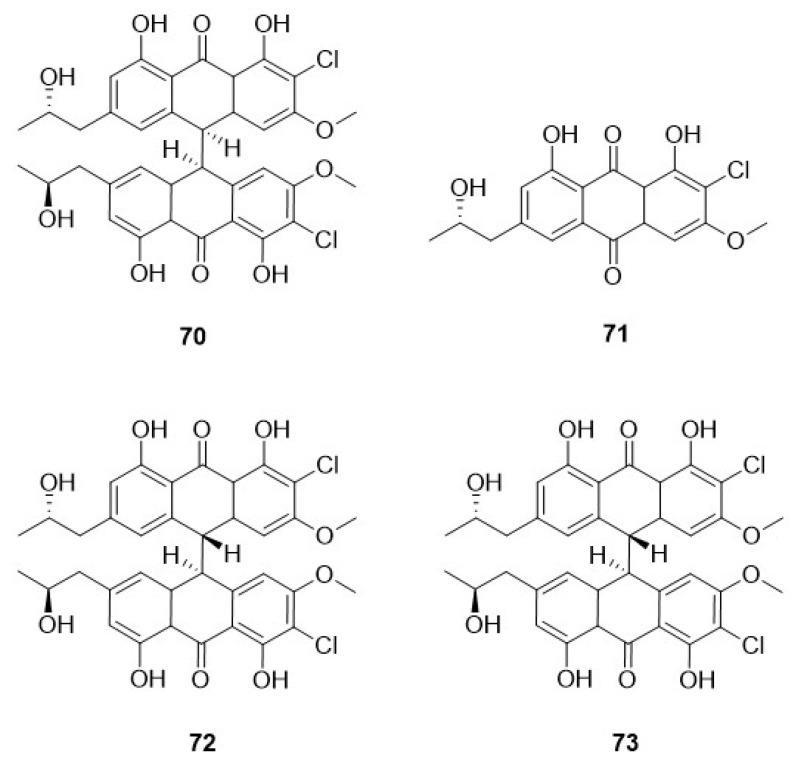
Chemical structures of Allianthrone A (**70**), Nalgiolaxin (**71**), Allianthrone B (**72**), and Allianthrone C (**73**).

Mandelare et al. [227] describe the production of novel chlorinated bianthrones from the co-culture of two different fungal developmental stages; a seaweed-derived *Aspergillus alliaceus* in morph state and *Petromyces alliaceus* in teleomorph state. The vegetative stage (morpho asexual) can be separated from the one that goes on to sexual development (sclerotial morph); both produce distinct secondary metabolite patterns in monoculture, thus in the monoculture of the sclerotial morph was found mainly Ochratoxin, while the anthraquinone pigment Nalgiovensin was produced by the asexual morph. Their co-culture significantly changed the metabolic profile of the strain. The chlorinated compound Nalgiolaxin was abundant, and newly produced bianthrones were found. Allianthrone A and its two diastereomers were isolated. Allianthrone A showed weak cytotoxicity against HCT-116 colon cancer and SK-Mel-5 melanoma cell lines (Figure 10).

**Figure 11:**
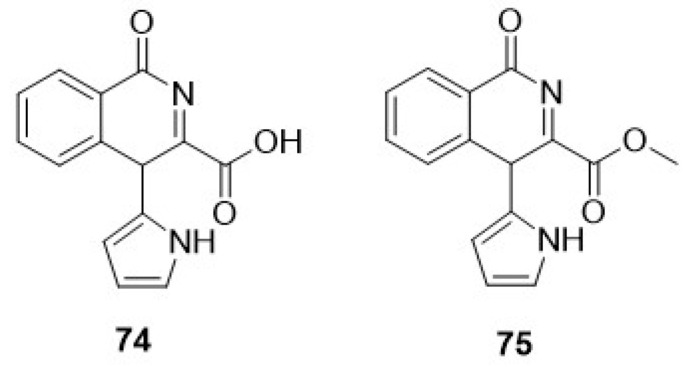
Chemical structures of Marinamide (**74**) and its methyl ester (**75**).

Using the mixed fermentation technique of two endophytic mangrove fungi (strain Nos. 1924 and 3893) from the South China Sea, Zhu and Lin [228] produced a new alkaloid, 1-isoquinolone analog, named as Marinamide (**74**) and its methyl ester (**75**) (Figure 11).

When strains were grown in isolated cultures under the same conditions, compounds **74** and **75** were not obtained. However, results obtained when the mixed fermentation technique was used showed that this technique may represent a potentially important approach for new metabolites.

As Edrada-Ebel et al. indicate in their conclusions, it is not easy to make generalizations about the expected results of certain co-cultivation techniques. On the other hand, it should be noted that other culture techniques could be associated with mixed fermentation methods, such as I-chip [229]. I-chip is an in situ culture technique that uses a device for the isolation of bacteria that are difficult to culture in the laboratory. This device contains hundreds of miniaturized diffusion chambers that are inoculated with a single bacterial cell. This device can be placed in the natural environment of the organisms cultured in the diffusion chambers, either in sediment, soil, or macroorganisms, as has recently been performed in a sponge (*Xestospongia muta*) [230]. I-chip allows microorganisms to grow in their natural environment, it also allows microbial interactions. The I-chip strategy leads to the discovery of new organisms that are able to provide new molecules as antibiotics [229].

Although screening of secondary metabolites is usually performed using single-strain cultures, due to, among other reasons, the lack of biotic and abiotic interactions in monocultures, growth conditions are very different from those in a natural environment, reducing diversity and forcing frequent re-isolations of known compounds. In recent years, several methods are being developed to understand the physiological conditions under which cryptic microbial genes are activated, to stimulate their biosynthesis and trigger the production of hitherto unexpressed chemical diversity.

### 5.2. Ex Vivo Biosynthesis or In Vitro Multienzyme Synthesis

One emergent and powerful approach for drug development and optimization implies the use of an in vitro multi-enzyme synthesis [14,231,232]. The highly regio- and stereospecific nature of enzymatic catalysis facilitates the assembly of complex biomolecules through the activities of various enzymes in a single reactor which is usually impossible in conventional chemical syntheses [4,233,234,235].

In contrast to whole organism engineering, which does not provide insights into the mechanisms of individual biosynthetic enzymes and requires knowledge of discrete intermediates, in vitro multienzyme synthesis overcomes issues related to metabolic trafficking and growth conditions without the complications associated with whole cell transformations like uptake, metabolism, or toxicity [236]. It is a valuable alternative to precursor-directed biosynthesis using mutant bacteria, allowing for greater control over substrate and enzyme interactions.

Recent advancements indicate that there are virtually no limits to the number of enzymes that can be combined in a single reactor to produce complex structures with good yields. Combining designer enzymes in a multistep cascade reaction eliminates the need for purification steps, recycles expensive cofactors, and couples favorable and unfavorable reactions. Remarkably, the lack of product/substrate inhibition, likely due to the irreversible nature of many later steps in a biosynthetic sequence, enhances the efficiency of these syntheses. This enzymatic approach also avoids the unwanted degradation of unstable intermediates, in addition to the minimal use of hazardous chemicals and minimal production of waste [237].

However, in vitro multienzyme synthesis requires the isolation and characterization of the full set of biosynthetic enzymes and, sometimes, expensive cofactors such as nucleoside triphosphates, NAD(P)^+^, S-adenosylmethionine (SAM), or 3′-phosphoadenosine-S-phosphosulfate (PAPS) are required. In these cases, cofactors can be regenerated with a coupled enzyme system [233,238].

The field is poised to expand into the synthesis of natural products that are currently inaccessible through conventional chemical synthesis [34,239]. The examples collected in this section try to highlight the potential of biocatalytic methods in the production of complex natural compounds through a minimal number of enzymatic steps, illustrating the power of modern biocatalytic strategies, particularly in the context of MNPs that often present significant synthetic challenges, demonstrating the remarkable capabilities of multienzymatic synthesis [240].

There have been notable reports of de novo syntheses of complex products from simplified building blocks such as Tetracenomycin and other aromatic polyketides [241,242,243], that often require elaborate organic synthesis due to their complex chemical structure. The ability to generate these products in vitro not only enhances our understanding of the underlying biosynthetic mechanisms but also provides a platform for the development of novel therapeutic agents derived from these compounds thanks to their biosynthetic enzymes which serve as powerful biocatalysts in various transformation, including oxidation, glycosylation, and macrocyclization reactions.

The first in vitro assembly of a complete type II polyketide synthase (PKS) enzymatic pathway to natural products is the multienzyme total synthesis of the marine bacteriostatic agents Enterocin (**76**) and Wailupemycin A (**77**) y B (**78**) from *Streptomyces maritimus* and it represents a significant advancement in the field of natural product synthesis [244]. This synthesis, developed in a single reaction vessel, involved nine recombinant proteins and three commercial enzymes and started from simple benzoate and malonate substrates. It yielded approximately 25% overall and consumed seven equivalents of malonyl-CoA and two equivalents of NADPH per molecule of benzoic acid, along with S-adenosylmethionine (SAM) and ATP [244].

The successful one-pot, total enzymatic synthesis of twenty-four Wailupemycin and Enterocin analogs, many of which are novel chemical entities, highlights the potential of multienzyme systems to expand the functional nature of biosynthetic pathways [245].

Three years later from Enterocin (**76**) and Wailupemycin’synthesis, an efficient one-pot enzymatic synthesis of the anthraquinone antibiotic Rabelomycin (**79**), a member of the angucycline family, was successfully accomplished, starting from the natural building blocks acetyl-CoA and malonyl-CoA and utilizing a mixture of polyketide synthase (PKS) enzymes derived from the biosynthetic pathways of Gilvocarcin, Ravidomycin, and Jadomycin [246]. Angucyclines form the largest group of aromatic polyketides. They feature a tetracyclic benz[α]anthracene skeleton and have a broad range of biological activities including antitumor, cytostatic, enzyme inhibition, antibacterial, antiviral, and inhibition of platelet aggregation function [247].

Starting from the same natural building blocks as Rabelomycin (**79**), the successful one-pot enzymatic total synthesis of Defucogilvocarcin M (**80**), a notable polyketide intermediate from the gilvocarcin family, represents a significant milestone. This process is the longest reported sequence of enzymatic total synthesis, involving 15 enzymes—including oxygenases and a 2,3-dehydratase—from various sources, all expressed in *Escherichia coli* and purified as His6-tagged proteins [248]. Although no evidence has been found in the scientific literature indicating the isolation of gilvocarcins, such as Defucogilvocarcin M (**80**), from marine sources, this example has been included in this section due to the large number of enzymes involved in its biosynthesis. This complex biosynthetic system is not only significant for understanding natural metabolic pathways but also provides valuable perspectives for designing new strategies in combinatorial biosynthesis. These tools have the potential to inspire and develop innovative synthetic routes applicable to marine natural products (MNP), thereby expanding the possibilities for research and production of bioactive compounds. Notably, research has suggested that three oxygenases—GilOI, GilOII, and GilOIV—may form a multienzyme complex responsible for a concerted reaction pathway that includes crucial carbon-carbon bond cleavage, leading to the formation of the Gilvocarcin scaffold. Subsequent steps in the pathway involve methylation of aromatic hydroxy groups, formation of a hemiacetal ring, decarboxylation, and C-glycosyltransfer to produce pregilvocarcins. These intermediates are further converted into the final lactone-containing products by the enzyme GilR. Additionally, combinatorial biosynthetic enzymology revealed that only four enzymes—GilOII, GilM, GilMT, and GilR—were necessary to convert dehydrorabelomycin into Defucogilvocarcin M with a remarkable yield of 80% [248].

Also, it is possible to generate structural diversity and complexity using a limited set of catalysts as happens in the first total enzymatic synthesis of meroterpenoid natural products which was accomplished by Moore’s group. The enzymatic synthesis of Merochlorins A (**81**) and B, halogenated meroterpenoids with significant antibacterial activities, derived from the marine bacterium *Streptomyces* sp. [249] was achieved employing heterologously produced enzymes and began with simple substrates such as dimethylallyl diphosphate (DMAPP), geranyl diphosphate (GPP), and malonyl-CoA [112]. The process involved a combination of only four recombinant enzymes from the *mcl* biosynthetic machinery: type II polyketide synthases (Mcl17), a prenyl diphosphate synthase (Mcl22), an aromatic prenyl transferase (Mcl23), and a vanadium-dependent haloperoxidase (Mcl24) [112].

Another significant example is the biocatalytic total synthesis of Ikarugamycin (**82**), a polyciclic tetramate macrolactam which exhibits anti-tumor activities on several cancer cell lines [250,251]. Thanks to an efficient one-pot reaction with only three recombinant enzymes, specifically the PKS/NRPS machinery system (IkaA) along with two reductases (IkaB and IkaC), the product was isolated in 9% yield [252]. The iterative PKS/NRPS system (IkaA) played a central role, stitching together two hexaketidic polyene precursors derived from acetyl-CoA and malonyl-CoA and facilitating the attachment to *L*-ornithine. The final cyclization to form the tetramic acid moiety occurred with the help of IkaB and IkaC, which established the stereocenters in the carbocyclic structure of Ikarugamycin [252].

Traditional total synthesis methods for Ikarugamycin (**82**) have been labor-intensive and inefficient, often requiring 27 to 32 individual synthetic steps, resulting in overall yields of less than 1% [252,253,254,255]. This stark contrast underscores the advantages of biocatalysis, which not only simplifies the synthetic route but also enhances the efficiency and selectivity of the process.

**Scheme 21:**
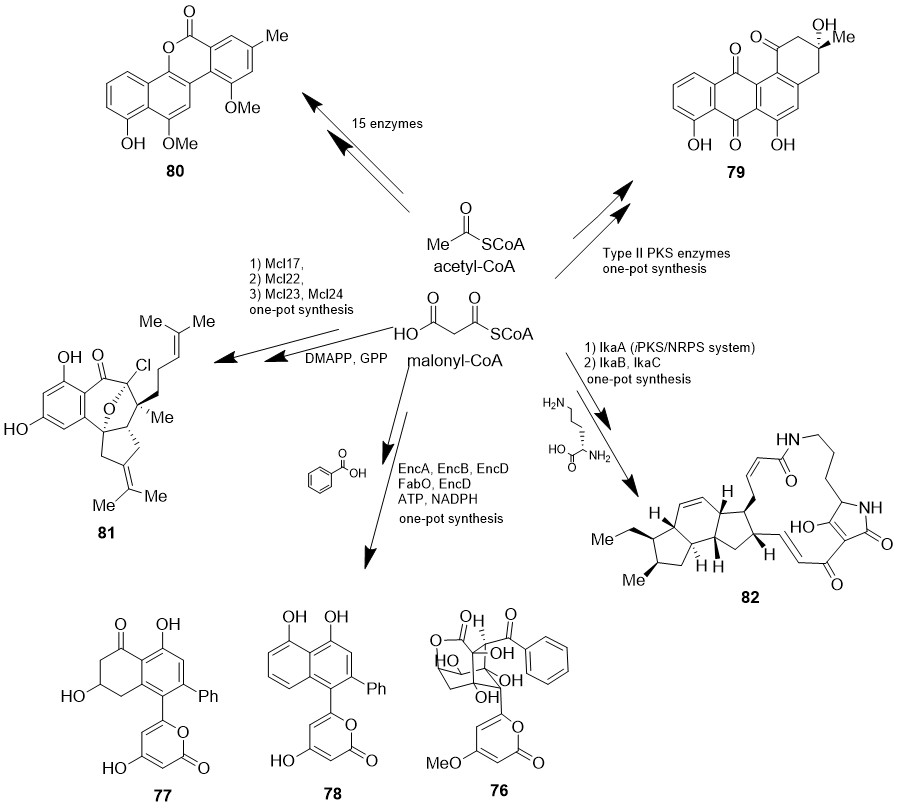
Schematic representation of total enzymatic synthesis of complex products from simplified building blocks: Enterocin (**76**), Wailupemycin F (**77**), Waylupemycin G (**78**), Rabelomycin (**79**), Defucogilvocarcin M (**80**), Merochlorin A (**81**), and Ikarugamycin (**82**). The diagram illustrates the stepwise enzymatic conversion of precursor molecules (acetyl CoA y malonyl CoA) into final products. Cofactors and reaction conditions are omitted for clarity.

These examples demonstrate the potential of total enzymatic synthesis in the large-scale production of marine-derived pharmaceuticals. By leveraging natural biosynthetic pathways and optimizing enzyme catalysis, researchers have developed sustainable, efficient methods for producing complex marine drugs (Scheme 21). To move from milligram to gram-scale reactions and beyond, some groups envisioned transitioning from in vitro reactions, conducted with purified proteins, to a scalable platform that would be readily accessible to synthetic chemists, using whole cells containing heterologously expressed proteins [120]. In a lyophilized form, cells can be easily weighed and directly used in reactions without significant loss of enzymatic activity compared to fresh wet cells [176]. This production strategy will be analyzed in the next section of the article.

### 5.3. Heterologous Expression of Biosynthetic Gene Clusters (BGCs)

Marine natural products, given their complex and novel structures, are an interesting resource to develop as leads for new drugs. However, in marine drug research, there are some difficulties that could be considered as bottlenecks: their complex structures make it difficult to synthesize them in sufficient quantity to carry out studies on drug validation and structure optimization. Even when a sample proves to be active during its first screening, due to the difficulty of obtaining marine samples in a reproducible way and considering the extremely low content of active components in these samples, it is a huge task to obtain a sufficient mass of marine natural products to allow a thorough evaluation of their biological activities and medicinal properties. Furthermore, there are many difficulties and challenges to be overcome to achieve yields that will make the drugs available in the quantities needed for commercialization. Fortunately, the rapid development of bioinformatics together with genetic engineering tools allows the identification of biosynthetic gene clusters in the genome of microorganisms [192] that offer us the possibility of designing new routes to produce and modify marine natural products. New biotechnology techniques make possible the production of new drugs from marine natural products. The reconstruction of biosynthetic pathways in heterologous hosts to create an MNP cell factory requires in-depth knowledge of the enzymatic reactions involved in the biosynthesis of the target compound in native host organisms [20]. It is true that the quantities usually obtained are not the desired ones, although the efficiency of these natural marine products has been significantly increased.

Biosynthetic gene clusters, BGCs, are the genetic material required to synthesize a microbial secondary metabolite.

**Figure 12:**
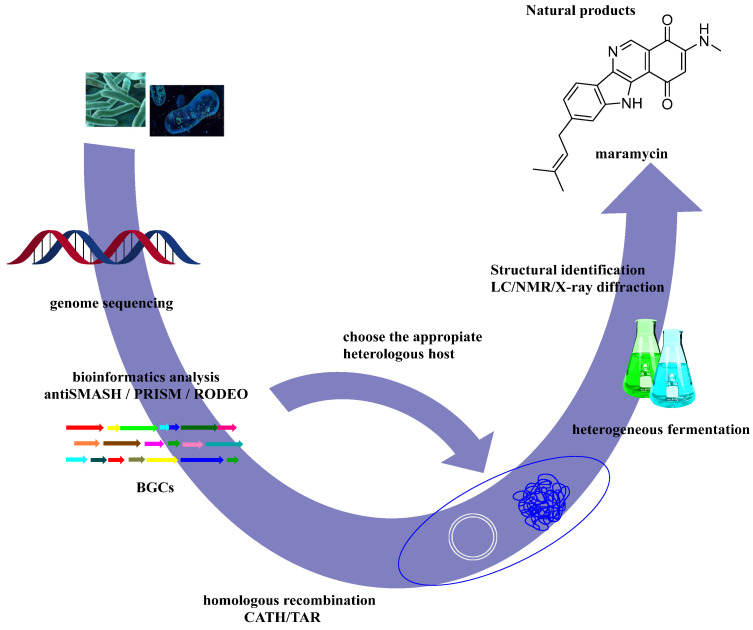
Summary of the general strategy of heterologous biosynthesis.

Figure 12 shows the strategy typically followed for heterologous expression of biosynthetic gene clusters (BGCs): first, DNA is extracted from the original host and genome sequences are obtained. Then, with the help of bioinformatics tools such as antiSMASH 7.0 [256], PRISM [257], and RODEO [258], the BGCs are determined. Finally, the BGCs are cloned and assembled into expression plasmids using appropriate methods such as TAR (transformation-associated recombination) [259] or CATCH (Cas9-assisted targeting of chromosome segments) [260,261] used to increase the speed of this step. The prepared recombinant plasmids are transformed into a heterologous host for expression. Once the host successfully expresses BGC, the culture result is examined by liquid chromatography-mass spectrometry (LC-MS) and other analytical methods to determine whether the heterologous host produces the desired secondary metabolites or not. Research on this topic has succeeded in assembling a large volume of genomic sequences from the ocean and has uncovered many new BGCs. These discoveries offer great potential for the identification of new MNPs and have renewed interest in isolating MNPs from complex marine resources. However, the cryptic culture conditions of marine microorganisms limit their studies and applications [262]. Nevertheless, deep sequencing analyses of amplicons suggest substantial biosynthetic potential of non-culturable bacteria. Since the original hosts cannot be used directly, heterologs, which are easier to grow in the laboratory, can be used to synthesize MNPs.

Genome mining combines several molecular biology techniques, such as DNA sequencing and bioinformatics with the aim of gathering information about the Biological Gene Clusters of the organisms and predicting the physicochemical properties of the products synthesized from BGCs. The ability to connect natural product compounds to BGCs, and vice versa, along with the increasing knowledge of biosynthetic machinery has given rise to the field of genomics-guided natural products genome mining of natural products, for the rational discovery of new chemical entities. In the field of natural product genome mining, it is important to find ways to rapidly link orphan biosynthetic genes to their associated biosynthetic mechanisms. This validates the enormous potential of these BGCs in synthetic biology applications. The clustering of specialized genetic elements brings an important advantage like the ability to clone and transfer whole BGCs into heterologous host organisms, such as bacterium and/or yeast for expression to allow de novo synthesis of the expected drugs by the transformed microbe and characterization. Nevertheless, in the heterologous expression of marine natural products, it is crucial to consider the heterologous host selection as well as genetic manipulation for BGCs. Xu et al. [263] review the problems around heterologous expression of MNPs from marine BGCs that focuses on two in two steps: (1) good selection of heterologous hosts and (2) genetic manipulations for BGCs. In this work, they categorized heterologous hosts into four groups: model strains of *Streptomyces* are of crucial importance in the biosynthesis of antimicrobial agents derived from actinomycetes. *Escherichia coli* is an ideal heterologous host for bacterial BGCs. Cyanobacterial model strains are ideal hosts for marine natural product bacterial BGCs, particularly for antimicrobial agents of all cyanobacterial species. *Aspergillus*, helps to express fungal MNP BGCs and the microalga *Fistulifera solaris*, for fungal BGCs. Xu et al. reported more than forty MNPs with titer among <1 mg/L for 4-*O*-demethylbarbamide (fatty amide) [264] and 668.97 mg/L for Beauvericin [265].

Mollemicin A (MOMA) is a unique glycohexadepsipeptide-polyketide isolated from a *Streptomyces* sp. from the Australian marine environment. MOMA exhibits remarkable inhibitory activity against both drug-sensitive and drug-resistant malaria parasite bacteria. The production titer of MOMA in the wild-type strain is low, so, optimization of MOMA by structural modifications or product improvements is necessary to obtain efficient analogs. In this regard, Jin et al. [266] have identified and characterized, for the first time, the biosynthetic gene cluster of MOMA, proposed its complex biosynthetic pathway and developed a new biosynthetic production. They achieved effective improvement of MOMA production by applying combined fermentation optimization and genetic engineering strategies. By optimizing the fermentation medium, the yield of MOMA increased from 0.9 mg L^−1^ to 1.3 mg L^−1^ (44% yield increase). Complementarily, they have developed a synergistic mutant strain by deleting the momB3 gene and overexpressing momB2, thus obtaining a 2.6-fold increase from 1.3 mg L^−1^ to 3.4 mg L^−1^. These findings allow the investigation of the biosynthetic mechanism of MOMA, while providing the possibility to produce a wide range of MOMA analogs as well as to develop an efficient strain for the sustainable and economical production of MOMA and some of its analogs.

Heterologous expression methodologies are well developed and are being used to increase the titer of known compounds of interest, to produce compounds derived from cryptic gene clusters or environmental DNA (eDNA) as the recent identification of biosynthetic gene clusters for terpene biosynthesis in the genomes of marine corals [267] and to produce analogs [263]. Heterologous expressions of BGCs for characterization or identification of new chemical entities are also advantageous for several reasons. As more sequences from diverse microbial sources become available, heterologous expression obviates the need to develop new genetic tools to probe the pathways of each new genus or species of interest. Successful heterologous reconstitution of a BGC allows relatively rapid design of all the essential specialized genes involved in the production of a microbial biochemical. So, it is possible to characterize BGCs from microbes that have not yet been cultured, such as those identified from obligatory symbionts or environmental DNA (eDNA).

An emerging and powerful strategy involves the identification of the first set of candidate genes through metabolomic and transcriptomic analyses, with several metabolomic approaches now enabling the accurate detection of potential intermediate reactions. Recent advances in molecular networks further enhance the processing of acquired metabolomic data, providing new opportunities for discovery [268]. Complementing these approaches, Zhang et al. have reviewed genetic platforms established for the heterologous expression of microbial natural products, which expand the potential for harnessing these biosynthetic pathways [195].

The main limitation of genome mining is that only known BGCs can be recognized [269], and it is unable to predict all biological activities of natural products. This methodology could be complemented by chemical structure identification techniques in the prediction of BGCs of interest.

Advances in biotechnology facilitate the development of a prototypical microbial strain capable of producing μg or mg per liter, where optimization of MNP synthesis in the microbial host to industrial scale currently represents a challenge where all marine natural product research technologies converge. While global approaches provide the basic structure of the main compounds, the production of the most efficient bioactive molecules will require multiple iterative refinements using the Design–Build–Test–Learn cycle. Synthesis mining of biological natural products is, in fact, one of the main applications of systems biology. This systems engineering shows great potential in the transformation and optimization of the metabolic network of microbial cellular factories. In this sense, the modeling and optimization of networks based on “-omics” technologies are currently one of the pillars of optimization strategies for the efficient synthesis of MNPs in microbial cell factories.

The advances in technologies including “-omics” tools for improved biodiscovery and the uncovering of metabolic pipelines, robotics, microfluidics, quantum computing, profiling, analytical and computational biology techniques for fractionation, and the isolation of secondary metabolites from crude extracts have considerably increased the exploration of natural sources [32]. The integration of “-omics” approaches with focus on genomics, transcriptomics, and metabolomics, elucidates the complexity of gene regulatory networks and significantly helps our understanding of complex mechanisms involved in the expression of biosynthetic gene clusters encoding secondary metabolites [270].

Isoquinolinequinones represent an important family of natural alkaloids with profound biological activities. They are predominantly isolated from marine invertebrates, some examples are cytotoxic caulibugulones and perfragilins from the bryozoan *Caulibugula inermis* [271] and *Membranipora perfragilis* [272], antineoplastic cribrostatins from the sponge *Cribrochalina* sp. [273], and antimicrobial and anti-inflammatory renierones from the sponge *Reniera* sp. [274] and *Haliclona* sp. [275]. However, the recent discovery of such compounds including the mansouramycins [276,277,278,279] and albumycin [280] from *Streptomyces* showed that microbes might be the true isoquinolinequinone producers opening the door to their production in microorganisms thanks to a heterologous expression of BGCs.

**Figure 13:**
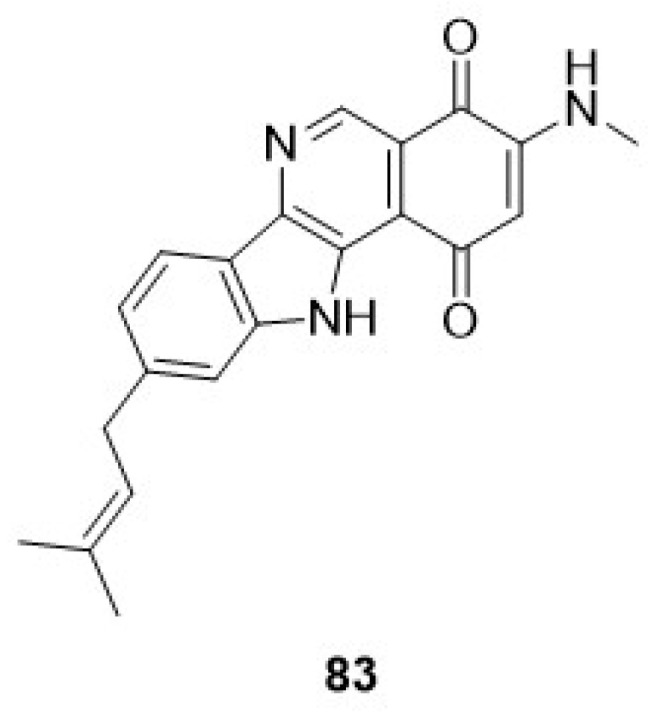
Chemical structure of Maramycin (**83**).

Specifically, Maramycin (**83**) has been recently discovered and characterized by Malekis et al. [281]. It is a new alkaloid with promising anticancer activity (Figure 13). The production of this compound arose from the crosstalk of a rare bifunctional prenyltransferase/tryptophan indolylase enzyme, which allows the generation of prenylated indoles from tryptophan, and the native biosynthesis of Mansouramycin. The authors believed that this enzyme may be useful for rationally designing tryptophan-derived natural products and creating promising new analogs. *Streptomyces mirabilis* P8-A2, produced azodyrecins a cytotoxic azoxy metabolites.

Heterologous expression of a rare bifunctional indole prenyltransferase/tryptophan indole-lyase enzyme from *Streptomyces mirabilis* P8-A2 in *Streptomyces albidoflavus* J1074 led to the activation of a putative isoquinolinequinone biosynthetic gene cluster and production of a novel isoquinolinequinone alkaloid, named Maramycin (**83**). The structure of Maramycin (**83**) was determined by analysis of spectroscopic (1D/2D NMR) and MS spectrometric data. The prevalence of this bifunctional biosynthetic enzyme was explored and found to be a recent evolutionary event with only a few representatives in nature. Maramycin (**83**) exhibited moderate cytotoxicity against human prostate cancer cell lines, LNCaP and C4−2B. The discovery of Maramycin (**83**) enriched the chemical diversity of natural isoquinolinequinones. Its production by heterologous expression shows the promise of this strategy which combines host and heterologous biosynthetic genes allowing the generation of new chemical scaffolds [239].

### 5.4. Combinatorial Biosynthesis and Metabolic Engineering

Combinatorial biosynthesis is an approach to engineering natural product (NP) biosynthesis where the biosynthetic pathway encoded by a BGC of interest is designed based on the substrate promiscuity of the enzymes involved to produce NP analogs. As is known, the specific functions of biosynthetic enzymes can be assigned to the genes or gene segments encoding the enzymes. This fact can be used so that such genes and gene segments can be mixed or manipulated to generate engineered enzymes that can produce NP analogs [282].

Combinatorial biosynthesis can include several approaches, such as metabolic engineering of microbes, modification of enzyme-encoding genes, and provision of small molecules as non-native components of biosynthesized products to generate a large library of products with novel chemical structures. Combinatorial biosynthesis can be achieved by engineering biosynthetic enzymes, particularly those that assume a modular organization, such as the polyketides (PKSs) and the non-ribosomal peptides (NRPSs.)

Several combinatorial biosynthesis methods are currently applied with the aim of creating a library of NP analogs. These methods can be classified into (1) precursor-directed biosynthesis where an alternative precursor molecule to initiate the biosynthetic steps is introduced to form a modified product, (2) mutasynthesis in which the incorporation of the wild-type precursor is blocked by inactivating a biosynthetic enzyme and an alternative pathway intermediate is provided to generate an altered product and (3) activation of silent gene clusters pathways with suitable promoters. Despite a late start, combinatorial biosynthesis has received much attention in the field of MNPs and has been used successfully in recent years [193].

Metabolic engineering describes that organisms can be explored at different levels, from the genome to the metabolome, through the transcriptome and proteome. Bertrand et al. summarize various strategies to increase the diversity of metabolites obtained from microbes with special emphasis on the multiple methods of performing co-culture experiments. In addition, they review the various analytical approaches to address the study of these interaction phenomena and describe the chemical diversity and biological activity observed among the induced metabolites [190]. At the genome level, one of the first techniques to be exploited was metabolic engineering, or “the process of redirection of metabolic pathways through genetic manipulation” as it was defined by Khosla and Keasling [283]. In recent years, metabolic engineering has enabled the development of several genetic manipulation techniques, the most common of which involves the biosynthesis of secondary metabolites in a heterologous host.

For example, in the case of a valuable MNP produced by a holobiont such as a marine invertebrate associated with several microorganisms, the investigation is tremendously challenging. The elucidation of the complete biosynthetic pathway requires the identification of highly specific enzymes encoded by genes present in multiple species and belonging to different superfamilies containing several tens to hundreds of members such as, for example, oxidases, reductases, methyltransferases, and cytochrome P450. The emergence in recent years of massive omics (genomics, transcriptomics, proteomics, and metabolomics) resources and tools could help to accelerate this research. At this stage, the availability of the holobiont metagenome and massive transcriptomic data is of vital importance, as the search for specific conserved functional domains can lead to defining the first sets of candidate genes. The most powerful and comprehensive approach to rationally prioritize gene sequences in metabolic engineering is based on gene co-expression analysis. Next, validation of the correct activity of each of the predicted genes/enzymes is required. For this purpose, biochemical characterization of recombinant proteins heterologously expressed in bacteria or yeast remains a basic but reliable approach. A key factor in achieving pathway reconstruction in a microbial chassis is the choice of an appropriate heterologous host (such as bacteria or yeast). Extensive synthetic biology tools are now available for both microbial models and integrative genomic constructs, including CRISPR-Cas9 technologies, and self-replicating plasmids. Once the gene transfer in the heterologous host has been performed, the main strategies for MNP synthesis in the microbial host include the optimization of media and culture conditions, but also the implementation of bioreactors, screening of enzyme variants with improved activities, adaptation of the host metabolism, and optimization of the enzyme target. CRISPR-Cas9 technology has also been used to knock out genes encoding well-known and often rediscovered antibiotics in several actinomycete strains, resulting in the production of different rare and unknown variants of antibiotics that would otherwise not have been discovered, such as Amicetin, Thiolactomycin, Phenanthroviridin and 5-chloro-3-formylindole [284].

Thanks to the application of the strategies and techniques of metabolic engineering, Pyne et al. [145] developed a yeast platform for the synthesis of tetrahydroisoquinoline alkaloids on a gram per liter scale, reporting in 2020 the production of Trabectedin (**29**).

**Figure 14:**
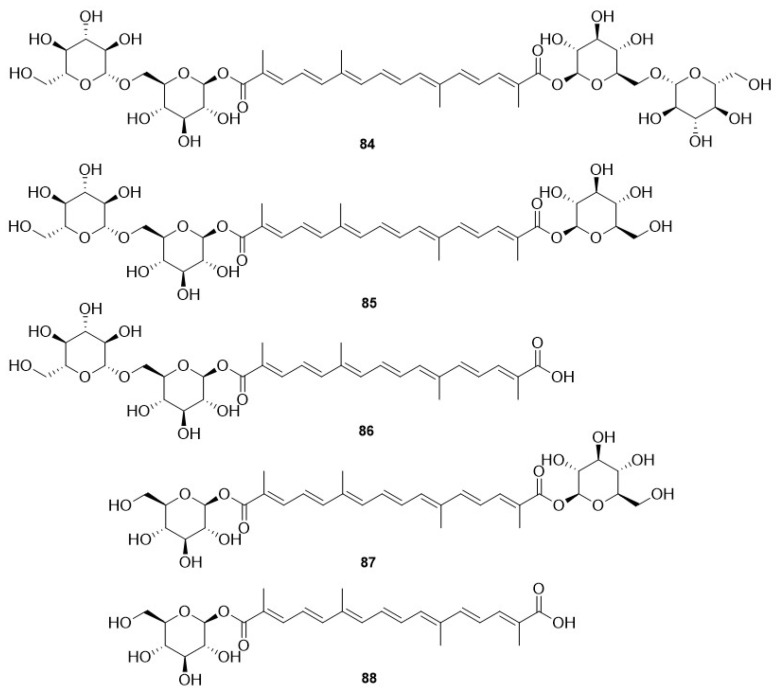
Crocin derivatives depending on the different glucose groups: Crocin I (**84**), Crocin II (**85**), Crocin III (**86**), Crocin IV (**87**), and Crocin V (**88**).

Another interesting example is related to Crocin, a well-known aromatic substance produced by plants as a secondary metabolite. While crocin is not of marine origin, the heterologous production of crocin using microorganisms represents a highly promising and commercially viable approach. It is a carotenoid-derived natural product found in the stigma of *Crocus* spp. which is usually stabilized by esterification with gentiobiose, glucose or other common sugar [285] (Figure 14). Crocin is a natural product widely used in the cosmetic, nutritional and pharmaceutical industry. As a medicine it is used as an anticancer agent, antioxidation, antihypertension, to reduce the risk of atherosclerosis and to prevent Alzheimer’s disease. Carotenoid biosynthesis pathway is the way in which Crocin biosynthesis occurs naturally in *Crocus sativus*. But given the long time required for its production, biosynthetic methods are being developed to obtain it in greater quantities. A detailed survey of recent progress in Crocin biosynthesis in various host cells has been carried out by Zhou et al. [286]. They also discuss the future development of crocin-related products using genetic engineering technology.

Chai et al. [287] successfully introduced Ps-CrtZ, Cs-CCD2, and Sca-LD genes into *Saccharomyces cerevisiae*, with a yield of 6.278 mg/L crocetin per fermentation resulting in the highest heterologous yield on record.

Wang et al. [288] introduced Cs-CCD2L and different UGTs genes into *Escherichia coli* and synthesized crocetin resulting in a yield of 4.42 mg/L. Xie et al. [289] transformed for the first time Gj-ALDH2C3 and the glycosyltransferase genes Gj-UGT74F8 and Gj-UGT94E13 in *Nicotiana benthamiana* (in higher plant), which to our knowledge, represents the largest heterologous synthesis of Crocin on record with a yield of 105.8945 mg/g DW of crocetins, of which crocetin I and crocetin II accounted for 99%. These cases provide solid support for the research of further heterologous production of Crocin.

Currently, there are reports of heterologous Crocin production in *Escherichia coli*, *Saccharomyces cerevisiae*, microalgae, and plants that do not naturally produce Crocin. Microalgae, microscopic photosynthetic eukaryotes living in aquatic environments, are characterized by a rapid growth rate, relatively easy modification of endogenous metabolic pathways, and a complement of silent or expressed genes in the aquatic environment. These facts could reduce the complexity of its metabolic engineering for use as a Crocin bioreactor [290]. The microalga *Dunaliella salina* is one of the most widely employed algal species for the commercial production of β-carotene. As it naturally produces high levels of β-carotene which is the substrate for Crocin biosynthesis, it is being analyzed in order to design a Crocin cell factory [291]. The growth of *D. salina* in a high salinity culture environment is an additional advantage as it can effectively inhibit contamination by other micro-organisms, which could have benefits in terms of reduced culture costs. Actually, Crocin production by *D. salina* as host is still in the initial stages. Although comprehensive and thorough research methods exist for using microalgae in the production of other carotenoid products, they are not sufficient to identify and modify key enzymes in the designed pathways and it will also require increased investment in the optimization of algal strains and further research and optimization of culture conditions as well as methods for exogenous genetic transformation and selection of transcription and translation-related factors [292].

In the field of medicinal chemistry, organofluorinated compounds are responsible for up to 15% of the pharmaceuticals on the market. Natural fluorinated molecules are very rare and, until now, the pharmaceutical industry has not had a microbial source of such compounds.

Although fluorinated molecules are commonly synthesized, the incorporation of fluorine into structurally complex natural products can be problematic. Most fluorination reagents are non-selective, harmful, and difficult to handle. To address these problems, precursor-directed biosynthesis and mutasynthesis have contributed to the generation of a host capable to synthesize fluorinated natural product derivatives in which simple synthetic organofluorine precursors are metabolized into more complex biosynthetic products [293]. As we have explained previously, the presence of halogen atoms in the structures of marine natural products, mainly chlorine and bromine, is a result of their concentration in the surrounding seawater and specialized halogenating enzymes in organisms [294].

In 2002, the fluorinase (5′-FDA synthase), which is responsible for the formation of the C-F bond formation in a rare bacterium *Streptomyces cattleya* which produces fluoroacetate and the amino acid 4-fluorothreonine, was discovered and subsequently characterized [295], opening up for the first time the possibility of using genetic engineering to produce fluorometabolites from fluoride ions in host organisms. It provides the opportunity to reprogram biosynthesis in a heterologous host for the assembly of an engineered organofluorine.

**Figure 15:**
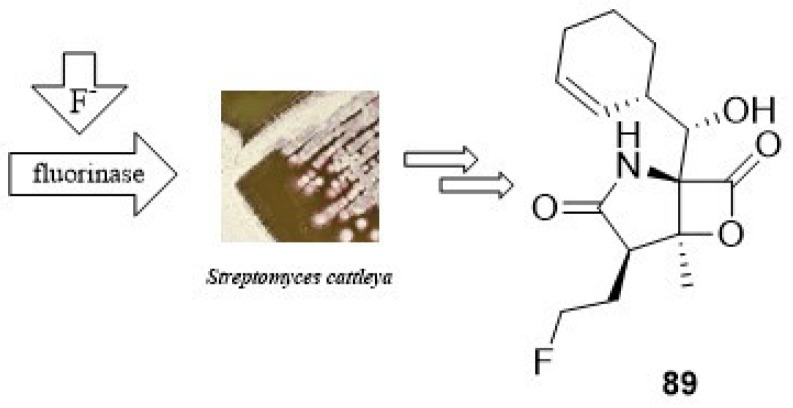
Fluorinase expression to production of Fluorosalinosporamide (**89**).

The marine bacterium *Salinispora tropica* was selected as the test organism, as it is the only one that biosynthesizes the potent anticancer agent Salinosporamide A from a chlorine halogenation pathway via 5′-ClDA in a reaction catalyzed by SalL-chlorinase (Figure 15) [296,297].

Eustáquio and co-workers [298] reported the induced production of fluoro-salinosporamide by substituting the gene for chlorinase gene salL from *Salinispora tropica* by the fluorinase gene flA. (yield 4 mg/L).

## 6. Computer-Aided Approaches to Development of Marine Natural Products

As mentioned in previous sections, microbes, microorganisms, and marine organisms, including some corals, represent a valuable source of secondary metabolites. These metabolites play a pivotal role in the discovery of new drugs. Pharmaceutical pipeline filling can be substantially improved by applying computational chemistry methods and tools. Although these techniques applied to marine drug lead discovery are still under development, significant advances have been reported. A Special Issue “Marine Drug Discovery through Computer-Aided Approaches” maps the main milestones of computer-assisted techniques in current blue biotechnology research [299].

The use of computational strategies can be a breakthrough given their potential to change and optimize the early stages of the drug development process, both in terms of time and cost reduction. Computational methods are important for identifying and elucidating the complex structures of marine natural products, predicting their chemical synthesis as well as identifying and validating biological targets and predicting their biological activities [300].

Given the enormous potential that computational chemistry methods represent in the investigation of MNPs, there are numerous studies that analyze from a chemical structure and electron density point of view the possible activity of such MNPs as potential drugs. As an example, Glossman-Mitnik et al. [301] have reported the results of pharmacokinetic analysis of Patellamides A-G (cyclic peptides produced by marine cyanobacteria with potential pharmaceutical properties) [302] including reactivity descriptors, biological targets, and ADMET parameters by Conceptual DFT based Computational Peptidology (CDFT-CP) approach. Conceptual Density Functional, also called chemical reactivity theory, enables us to find the relationship between the bioactivity and the chemical reactivity from a series of global and local descriptors introduced from the fundamentals of the method.

The Density Functional Theory (DFT) and the Conceptual Density Functional Theory (CDFT) are two closely related theoretical frameworks used in the field of quantum chemistry to understand and predict the electronic structure and properties of molecules and materials. Although they share common principles, they have different approaches and applications.

Thus, one of the main differences between DFT and CDFT is that DFT focuses primarily on the calculation of the electron density distribution of a system. It is a valuable tool for the calculation of total energy and various molecular properties based solely on the electron density. Thus, the Density Functional Theory (DFT) can describe in an efficient way the electronic structure of a system, being a fundamental tool for predicting molecular properties.

In contrast, the Conceptual Chemical Reactivity Theory (CDFT) is concerned with the conceptual aspects of chemical reactivity and bonding. In this sense, it employs a range of quantitative measures, including electronegativity, hardness, and softness, to describe the electronic structure of molecules and their reactivity. These measurements are founded upon the tenets of quantum mechanics, thereby enabling the prediction of the reactivity of molecules [303,304,305,306].

A recent study [307] employed a broad combination of in silico and in vitro methods to identify a promising new source of MNPs for further study and development. *Caulerpa racemosa* is a type of green algae known as green caviar. This organism holds great promise for use in medicine, especially in the study of cancer. Using computational modeling (in silico) and laboratory cellular experiments (in vitro), its chemical components were effectively analyzed and found to have potential for treating non-small cell lung cancer (NSCLC). The study put special emphasis on blocking SRC, STAT3, PIK3CA, MAPK1, EGFR, and JAK1 by molecular docking and in vitro assays. These proteins play a crucial role in the resistance pathway to EGFR tyrosine kinase inhibitors in NSCLC. The chemical Caulersin (C2) included in *C. racemosa* extract (CRE) has been identified as a potent and effective agent in the fight against NSCLC, both in silico and in vitro. CRE and C2 showed a similar level of inhibition as Osimertinib (positive control/NSCLC drug).

## 7. Artificial Intelligence in Marine Novel Drug Discovery

It is now known that Artificial Intelligence (AI) plays a crucial role in pharmaceutical research. Kumar [308] analyzes the current landscape and shows in his graphical overview in a clear way the different steps involved in artificial intelligence for drug design.

AI can be integrated into various phases of NP drug discovery and this intervention can help in marine drug discovery. At MIT, a team of researchers used the ML algorithm to identify a drug called Halicin that kills many strains of bacteria [309]. Along with the elucidation and discovery of biologically active molecules, AI can help in the evaluation of the activity, target prediction, toxicological prediction of marine drugs, and its role in drug repositioning studies [308]. Consequently, this will accelerate the drug discovery process through pharmacological synthesis of marine natural product derivatives. DSP-1181 (a long-acting, potent serotonin 5-HT1A receptor agonist) is the first example of a drug entering clinical trials using the AI method in less than 12 months, compared to 4 years using conventional methods.

In view of the considerable number of variables that exert an influence upon the process of fermentation, the utilization of mathematical modeling represents a highly valuable tool in the context of fermentation processes. The application of machine learning has enabled the extraction of data patterns and information [310], thereby facilitating the understanding of how to cluster nonlinear data, such as that obtained in fermentation processes [311,312,313]. Examples of such processes include the production of two antitumor benzoquinones using *Saccharomyces cerevisiae* by artificial neural network with genetic algorithm [314], and the fermentation of *Spirulina platensis* with *Lactobacillus platarum* and *Bacillus subtilis* using random center optimization [315]. Since machine learning techniques have not been previously used in the analysis of experimental data on solid-state fermentation for alginate production using filamentous fungus, De Farias Silva and co-workers have used machine learning to predict the alginate lyase production yield (volumetric production) using *Sargassum* macroalgae as substrate and *C. echinulata* as producer, using Artificial Neural Networks (ANNs) and Support Vector Machine (SVM) in the analysis of several fermentation variables, such as pH of the nutrient solution, substrate moisture, inoculum concentration, and addition of an inducer. According to the authors, the empirical approach described in their study is a viable alternative for modeling and optimizing fermentation processes since SVM guarantees good predictability and accuracy. Their study made it possible to describe the bioprocess in greater detail than had been done so far, despite the limitation of phenomenological details for solid-state enzymatic fermentation.

With the development of biotechnology, computational tools, and artificial intelligence, together with the efforts of the scientific world and industry, we believe that the vast treasure of aquatic micro-organisms will gradually be discovered and that marine natural products derived from these micro-organisms can provide innovative solutions to human health problems, while contributing to improve the quality of life and preserving biodiversity.

## 8. Conclusions

Marine natural products (MNPs) are a promising pool for the discovery of scaffolds with high structural diversity and various bioactivities that can be directly developed or used as starting points for optimization into novel drugs. For the development of MNP hits into leads and ultimately into successful drugs, chemical modification may be required. In addition, bringing a compound into clinical development requires a sustainable and economically viable supply of sufficient quantities of it, employing different production strategies as aquaculture, semi-synthesis, chemical synthesis, biosynthesis, or the development of synthetic analogs with more manageable properties.

Total chemical syntheses, in general, involve numerous steps, the use of expensive and polluting reagents in many cases, the application of capricious synthetic reactions, at a high human and time cost. In many cases, when the compounds to be synthesized are simple, they are feasible and allow a more accessible scale-up, as in the case of nucleoside derivatives such as Cytarabine (**1**), Nelarabine (**4**), or Fludarabine (**9**). However, when the molecules are particularly complex as in the case of many of the compounds extracted from marine ecosystems, it is difficult to obtain them through total chemical synthesis and, therefore, even more difficult to obtain them on a large scale, as many of these syntheses are led to very low yields after long synthetic routes consisting of a lot of steps. In this review, we have described the syntheses at industrial scale-up level of compounds such as Plitidepsin (**21**), Trabectedin (**29**), and Bryostatin-1 (**25**), whose synthesis yields allow them to be obtained at gram levels being capable of supplying the quantities necessary for their commercialization by pharmaceutical companies. It is likely that this robust supply process will remain a key strategy for the industrial-scale production of future MNP-derived drugs. However, it is important to acknowledge that this production process currently faces significant environmental and economic challenges, given the high demand for various reagents and the generally low yields achieved.

Likewise, we have emphasized semi-syntheses of Plitidepsin (**21**) or Trabectedin (**29**), which have allowed the scaling up of the production of these MNPs, combining the greatness of chemical and biological technologies.

Also, chemoenzymatic synthesis which exploits the selectivity and efficiency of enzymes, offers a sustainable and scalable approach to addressing the complexities associated with MNP production. As enzyme technology continues to evolve, chemoenzymatic methods are expected to become increasingly pivotal in the pharmaceutical industry. Despite its promise, several challenges hinder the widespread adoption of this strategy in marine pharmaceuticals synthesis. The discovery and optimization of suitable enzymes for specific reactions are time-consuming and complex processes. Additionally, scaling these methods from laboratory to industrial production involves overcoming issues such as enzyme stability, activity, labor-intensive protein purification, substrate solubility, and cofactor costs. Ongoing research in enzyme engineering, enzyme immobilization, novel screening techniques, enzyme mutagenesis, and the development of efficient chemoenzymatic cascades will be essential for unlocking the full potential of this technology and facilitating the market introduction of innovative marine-derived drugs.

On the other hand, biotechnology facilitates the targeted production of valuable biological compounds by various organisms under controlled growth conditions, utilizing well-designed bioreactors. Despite recent advancements, challenges persist in optimizing the cultivation techniques for certain marine organisms to make them more efficient and economically viable. Further genetic research is necessary, although significant progress has been made in recent years. The primary challenge lies in bridging the gap between the discovery of new and transgenic strains and their commercial application for producing natural marine drugs. The use of genetic engineering tools is crucial for thoroughly investigating high-productivity strains. Although these studies are in their initial phases, leveraging advanced genome editing tools holds considerable potential for future exploration. In addition to product development, further research is required to upgrade strains for enhanced productivity, maintain growth rates, and improve cell potential for survival under stressed conditions. Such studies will be essential for transitioning from laboratory experiments to large-scale industrial and commercial production.

Recent research on the ex vivo synthesis of natural products has demonstrated the feasibility of reconstructing complex multistep pathways making use of genetic engineering. Also, these reconstituted pathways have already enabled the production of ‘unnatural’ natural products related to the original compounds. In the future, these approaches may facilitate the synthesis of natural products that are currently inaccessible through conventional methods.

Also, heterologous expression of biosynthetic genes from the source organism in host organism allows for the de novo synthesis of the desired drugs by the transformed microbe. Thus, for example, after appropriate optimization, crocin can be produced by microorganisms in bioreactors providing the same or higher yields than plant extraction with a lower carbon footprint, using a less costly and more environmentally sustainable process.

A systems biology approach, integrated with advanced technologies such as genomics, transcriptomics, proteomics, metabolomics, and computational strategies, holds significant potential for innovating drug design and discovering improved drug candidates. Metabolic engineering, which emerged alongside the synthetic biology era in the early 2000s, allows microbial conversion of basic substrate to desired compounds to be optimized.

Total chemical synthesis, semi-synthesis, and combinatorial biosynthesis using an MNP as a starting point for analog generation and biosynthetic engineering modifying biosynthetic pathways of the producing organism will be of great importance for the development of MNPs into successful drugs, introducing greater diversity into natural products. Research on MNP biosynthesis, heterologous production, and metabolic engineering will contribute to the industrial production of a variety of MNPs which will not only bring enormous economic benefits, but also beneficial effects on human health

So, all the background research accumulated in the past decades that have allowed for the implementation of industrial production strategies for different classes of MNPs from highly diversified sources may combine to benefit the supply of new marine drugs. By continuing to refine and expand this integrated approach, we anticipate a new era of molecular design and synthesis that drives innovation across multiple scientific disciplines.

## Data Availability

Not applicable.

## Abbreviations

AI	Artificial intelligence
ALL	Acute lymphoblastic leukemia
ADC	Antibody drug conjugate
Ara-A	Arabinosyl adenine
Ara-C	Arabinosyl cytosine
Ara-G	Arabinosyl guanine
Ara-U	Arabinosyl uracil
BGC	Biosynthetic gene cluster
CAL B	Candida antarctica lipase B
Cbz	Carboxybenzyl
CDFT	Conceptual Density Functional Theory
CDFT-CP	Conceptual Density Functional Theory based Computational Peptidology
DCW	Dry cell weight
DFT	Density functional theory
EBA	Enterobacter aerogenes
Et-743	Trabectedin
F-ara-A	F-Arabinosyl adenosine
FDA	Food and Drug Administration
FDMO	FAD dependent monooxygenase
GMPs	Good Manufacturing Practices
HDMS	Hexamethyldisilazane
HPLC	High performance liquid chromatography
I-SMEL	In situ Marine moleculE Logger
MNPs	Marine Natural Products
NAD^+^	Nicotinamide adenine dinucleotide
NADP^+^	Nicotinamide adenine dinucleotide phosphate
NCS	(*S*)-Norcoclaurine Synthase
PCR	Protein C Reactive
PCT	Patent Cooperation Treaty
PSTs	Paralytic shellfish toxins
PKC	Protein Kinase C
RCM	Ring closing metathesis
SA	Salicylic acid
STS	Soft tissue sarcoma
TEMPO	2,2,6,6-tetramethylpiperidinyloxy
